# Supplementary material for: The mortality burden from COVID in low-income settings: evidence from verbal autopsies in India
Source: BMC Public Health. 2026 Jan 22;26:1567. doi: 10.1186/s12889-026-26215-9 (PMC13188735; doi:10.1186/s12889-026-26215-9)
Supplement: Supplementary file 1 — Supplementary Material 1. [file 12889_2026_26215_MOESM1_ESM.docx]

SUPPLEMENTAL MATERIALS

The Mortality Burden from COVID in Low-Income Settings:

Evidence from Verbal Autopsies in India

Anup Malani

# Methods

**CPHS**. *Strata*. CPHS samples randomly within strata. Strata are defined in two dimensions. One dimension is a cluster of similar districts within a state which CPHS calls a homogenous region. The second dimension is 5 categories of human settlement type and size (very large towns with over 200,000 households, large towns with 60,000 - 200,000 households, medium towns with 20,000-60,000 households, small towns with less than 20,000 households, and rural villages that the Indian Census distinguishes from towns). The last of these categories is called a rural strata; the remaining are called urban strata. In 2018-2021, there were 102 unique homogenous regions and 382 unique homogenous region x town size strata.

*Sampling in strata*. CPHS samples home differently in rural and urban strata ^1^. In each rural strata, CPHS randomly selects roughly 30 villages in that strata. The number of villages selected per strata grows over time, but weights are adjusted to account for changes in this count and ensure that estimates from the sample can be made population-representative. Within each chosen village, CPHS chooses a random starting point on the main street of the village and then engages in systematic sampling, attempting to survey every n-th house on that street until it is able to sample 16 houses in the village. The value of n ranges from 5 to 15 and is picked at random.

In each urban strata that exists, CPHS randomly picks at least one town in the strata. (Some larger town sizes are not present in some homogenous regions.) Again, the number of urban strata and towns selected grows over time, but weights are adjusted to ensure estimates from the sample can be made population representative. In each selected town CPHS randomly selects 21 Census Enumeration Blocks, each a cluster of 100-125 households defined by the Indian government for the purpose of its decennial census. Finally, within each census block, CPHS uses systematic sampling to select 16 households.

*CPHS survey*. The CPHS survey creates a roster of each current member of a household. It also records household member-level changes in that roster and the reasons for the change, including birth, death, immigration away from household, and immigration into the household. The sample of deceased individuals in the CPHS data are obtained from this roster.

For each member of the household or the household as a whole, as appropriate, the CPHS survey captures demographics, income, consumption and financial status including assets and debt. Demographics include age and sex, as well as religion and caste. Income data permit calculation of quartile of income distribution.

**Weights for living CPHS sample members**. Weights for the living sample in the CPHS are premised on the survey having employed a stratified design with random sampling within strata. A living respondent’s “sampling weight” is N(j,t)/n(j,t), where N(j,t) is the total population in strata j in month t, and n(j,t) is the size of the CPHS sample in strata j at time t. Strata are defined by location, urban status, and town size if location is urban (as explained in this Supplement).

One problem with these sampling weights is that not all CPHS sample households respond to each CPHS survey wave. To address non-response, CPHS provides “non-response weights” for each responding living sample member to account for non-responding living sample members. These non-response weights assume non-response is random within strata. The weight for a responding sample member in strata j at time t is M(j,t)/m(j,t), where M(j,t) is the total CPHS sample intended to be surveyed in strata j in month t and m(j,t) is the number of CPHS respondents in strata j in month t. We multiply the sampling weight by the CPHS non-response weight to obtain the “CPHS weight” of each living respondent in the CPHS in each month.

**Weights for deceased CPHS sample members**.  *Adjustment for under-inclusivity of deceased sample*. The VA sample only includes deaths reported in CPHS during 2018-2021. Because deaths are reported with a delay, the CPHS sample of deaths is under-inclusive. To address this, we estimate an adjustment factor that we use to scale population all-cause and cause-specific death rates each month from 2018-2020 to capture all deaths that would be reported in 32 months, if 32 months of reports were available. (We chose 32 months because, in our secondary measure of date of death, i.e., midpoint between surveys, no death is reported later than 32 months after it occurs. Moreover, in our primary measure of the date of death, the VA survey, over 98% of deaths reported within 48 months are reported within 32 months.) This adjustment factor for death rates in a given month is the ratio of (a) the number of deaths that would be reported after 32 months of reporting and (b) the number of deaths that would be reported in t months, where t is the number of months between the given month and December 2021, the last date of reporting for this study.

This adjustment factor is estimated in three steps. First, we isolate decedents whose date of death is in the first 16 months of the VA sample, i.e., Jan. 2018-April 2019, inclusive. Second, for each month we calculate the ratio of (a) the number of deaths that month that were reported within 32 months of occurrence and (b) the number of deaths that month that were reported within t months, where t ranges from 1 to 32. This results in 32 ratios per month of death, for 16 months. Third, we take averages across the first 16 months of death for each t, resulting in 32 averages of (a)/(b). These 32 averages are our adjustment factors.

Note that the reciprocal of these adjustment factors give the fraction of deaths that are reported in 32 months that are reported within t months, where t ranges from 1 to 32. We plot these factors, which sketch an empirical cumulative distribution function for time until a death is reported, to illustrate the degree of adjustment required for each t in Figure S1.

*VA non-response weights*. We create weights to account for the fact that not all households with a decedent reported to CPHS between 2018-2021 respond to the VA survey. These weights are constructed and evaluated in three steps. First, using the sample of all persons reported to CPHS as deceased from 2018-2021, we estimate a probit regression wherein the dependent variable is an indicator for whether the decedent’s household responded to the VA and the independent variables are a range of household-level variables (number of members, number of children, and average adult age; religion and caste; educational status, literacy, and employment status of head of household; possession of mobile phone; CPHS weights) and strata x rural status fixed effects and report the results as an unadjusted test of non-random VA response. These data are measured as of CPHS round 1 in 2022, when we executed the VA survey, or the first prior month in which we have such data from a household. If a household has multiple deceased members, the household is included multiple times in this regression. We present the results of this regression in column 2 of Table S1 below. (Column 1 replaces the strata x rural fixed effects with a rural dummy to illustrate that rural status correlates with VA non-response.) We report p-values for variables that predict the largest differences in propensity to respond to the VA and the extent of variation in response explained by strata fixed effects.

Second, we estimate predicted probabilities p(i) of VA response for each deceased person i’s household. We calculate propensity-score adjusted weights for each deceased person; these are 1/p(i) for a person whose household responded to the VA and 1/(1-p(i)) for a person whose household did not. We re-run the regression from step one with these weights and report the results as an adjusted test of non-random VA response. To validate our weights, we re-estimate the probit regression in step one, but employing the propensity score-adjusted weight for deceased persons from responding and non-responding households. We present the results of this regression in the third column of Table S1.

Third, we use the propensity score-adjusted weight for deceased persons whose households respond to the VA to adjust for non-response to the VA when calculating all-cause or cause-specific death counts and rates. We do not use the propensity score adjusted weights for deceased persons whose households did not respond to the CPHS because we do not have cause of death for those individuals.

**Alternative dates of death**. In the main text, we report results where the date of death is obtained from VA interviews. When those interviews do not produce a “viable” date of death that is before the date on which a death is reported to CPHS, we use an alternative measure that is the midpoint between when a death is reported to CPHS and the last date that the decedent’s household responded to a CPHS survey. Here we expound on the alternative measure and provide results if the alternative measure were used even when the VA survey provides a viable date of death.

The alternative date of death is premised on three facts: the CPHS survey is attempted on each sample household every four months, a decedent’s family may not be interviewed in the month in which the decedent dies, and the CPHS does not capture the exact date of death. These facts imply that the date on which a decedent is reported as passed to the CPHS, which we call the date of report (DOR), is later than the actual date of death. They also imply that the date of death is after the last time that a household is successfully surveyed by the CPHS, which we call the date of last survey (DOLS).

Because the date of CPHS surveys of a given household are independent of the date of the decedent’s death and CPHS surveys roughly the same number of households per month, our alternative measure of the date of death is the midpoint between DOLS and DOR. If the decedent died between those two surveys, the midpoint is an unbiased estimate of the date of death.

How this is implemented is that if a household responded to two consecutive surveys, then we assign the date of death to be DOR minus 2, because surveys are 4 months apart. If the household skipped a survey, then the midpoint is further back from the DOR. For example, if 1 survey is skipped, then the gap is 8 months, so the midpoint is DOR - 4. In general, the alternative measure is DOR - ((s+1) x 2), where s is the number of surveys the household skipped. The maximum gap between any two surveys in our sample is 64 months, and so all deaths are within 32 months of the DOR.

Figure S2 reports the fraction of deaths that are reported to CPHS within t months (i.e., t months or less) following a death under the alternative dating of deaths. The x-axis is the number of months t since a death occurred. The y-axis is the fraction of deaths captured in the VA survey.

Figure S3A and B reports cause-specific deaths and death rates, respectively, using the alternative coding of the date of death. The new coding of death shows that SARS-CoV-2 is not as large a relative cause of death in 2020-2021. The reason is not that there are fewer SARS-CoV-2 deaths with the new date. Rather, the explanation is that cardiovascular and other deaths that are attributed to 2018-2019 in the dating method used in the main text are pushed “forward” into 2020-2021 in the alternative dating in this Supplement. This can be seen by comparing the average level or rate of cardiovascular and “other” deaths in 2020-2021 compared to 2018-2019. This implies the mid-point between surveys tends to be later than the date of death reported by the decedent’s family.

# Results

##### **Table S1**. Balancing test and propensity score weight validation.


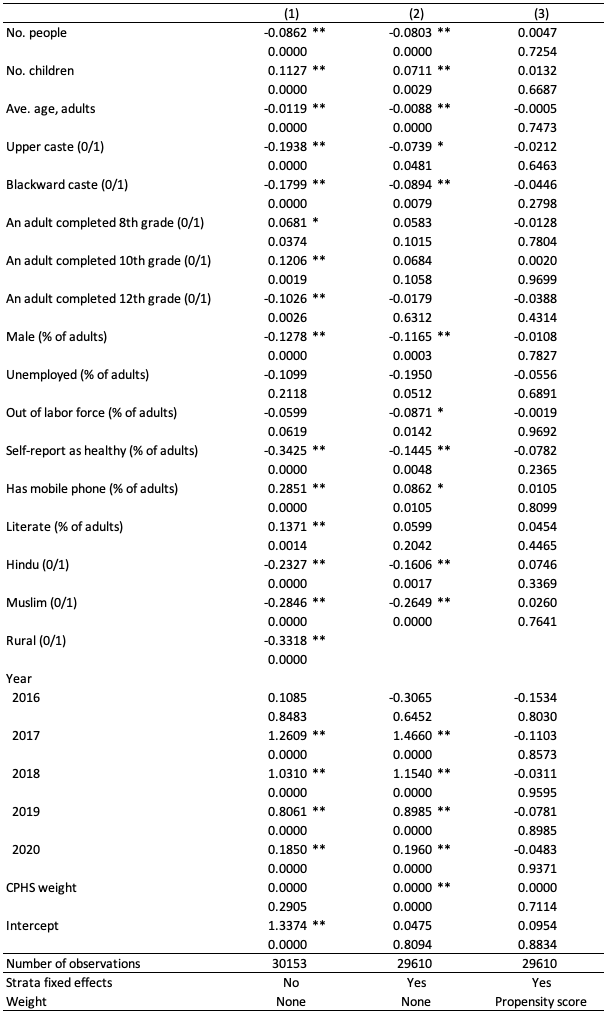


*Notes. Table reports results from probit regression of whether a household responded to an attempt to conduct a verbal autopsy survey on various household-level characteristics, including potentially location fixed effects. P-values are reported below coefficient estimates. Column 1 reports unweighted regression with a rural fixed effect but no strata fixed effects. Column 2 reports unweighted regression with CPHS strata fixed effects. Column 3 reports regression with propensity score weights equal to p for respondents and 1-p for non-respondents, where p is the predicted probability of responding from regression in column 2. There are 202 strata, defined as HR x urban status. HR means homogenous regions, defined as a cluster of districts within a state that are similar along a set of variables including agroclimatic condition.*

##### **Figure S1**. Delay in reporting deaths to the CPHS using date measurement in main text.

*
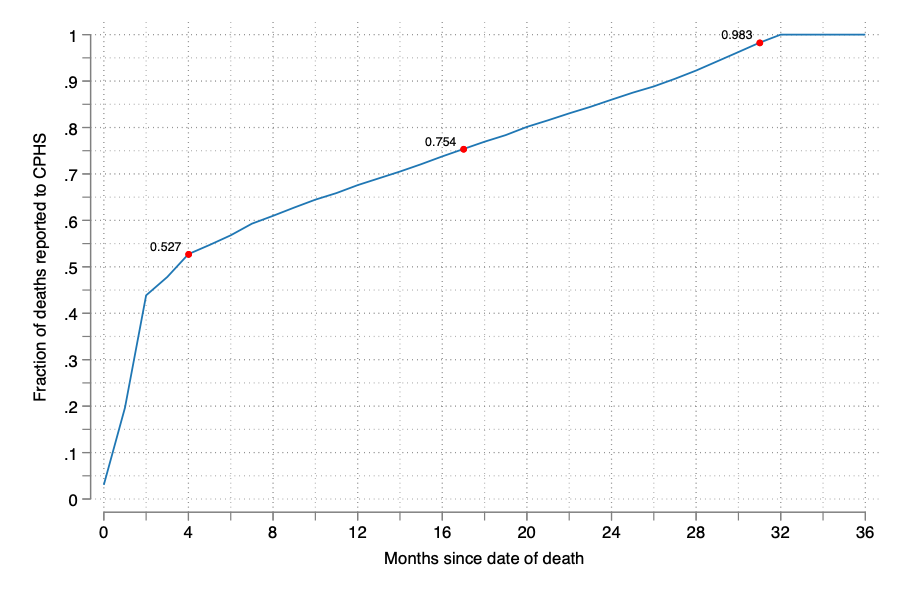
*

*Notes. This figure reports the fraction of deaths that occur in a month that are reported to the CPHS within the number of months indicated on the X-axis. The x-axis is the number of months after the date of death. Date of death is defined here as the date of death reported in the VA survey, unless that date is after the date the death is initially reported to the CPHS; if the date is after the CPHS report date, the date of death is the date that is the midpoint between the date that death is reported to the CPHS and the last date that the decedent’s household responded to a CPHS survey. The units of the y-axis are the fraction of deaths reported. The blue line gives the fraction that is the ratio of (a) the number of deaths reported in within t months of the date of death (numerator) and (b) the number of deaths reported within 32 months of the date of death.*

##### **Figure S2**. Delay in reporting deaths to the CPHS using alternative date measurement (mid-point between date of report and last completed survey).

*
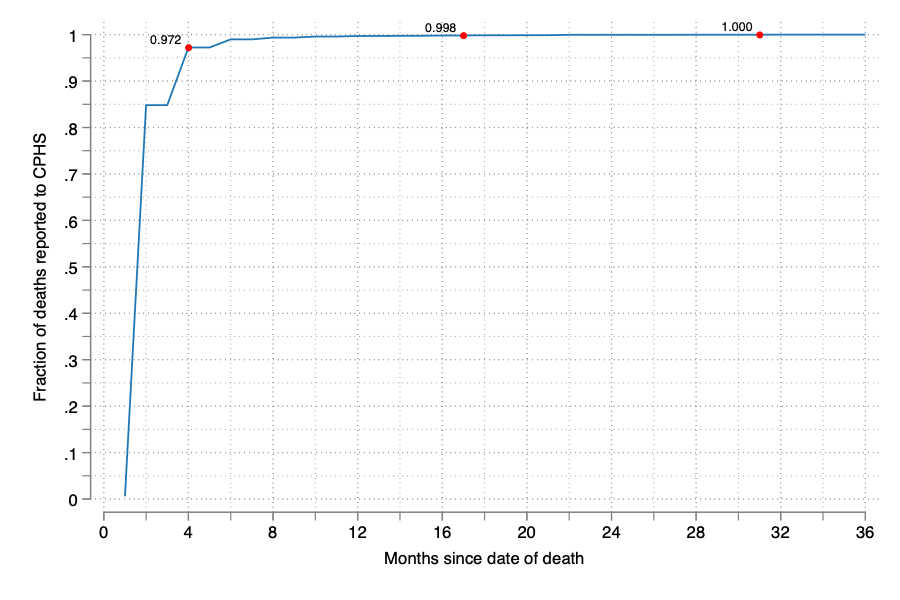
*

*Notes. This figure reports the fraction of deaths that occur in a month that are reported to the CPHS within the number of months indicated on the X-axis. The x-axis is the number of months after the date of death.* *Date of death is defined here as the midpoint between the date a death is reported to the CPHS and the date that the decedent’s household last completed a CPHS survey. The units of the y-axis are the fraction of deaths reported. The blue line gives the fraction that is the ratio of (a) the number of deaths reported in within t months of the date of death (numerator) and (b) the number of deaths reported within 32 months of the date of death.*

##### **Figure S3**. Monthly death counts for SARS-CoV-2, 5 other leading causes of death, and other deaths, 2018-2021, using the alternative date of death.

##### A: Deaths

#####
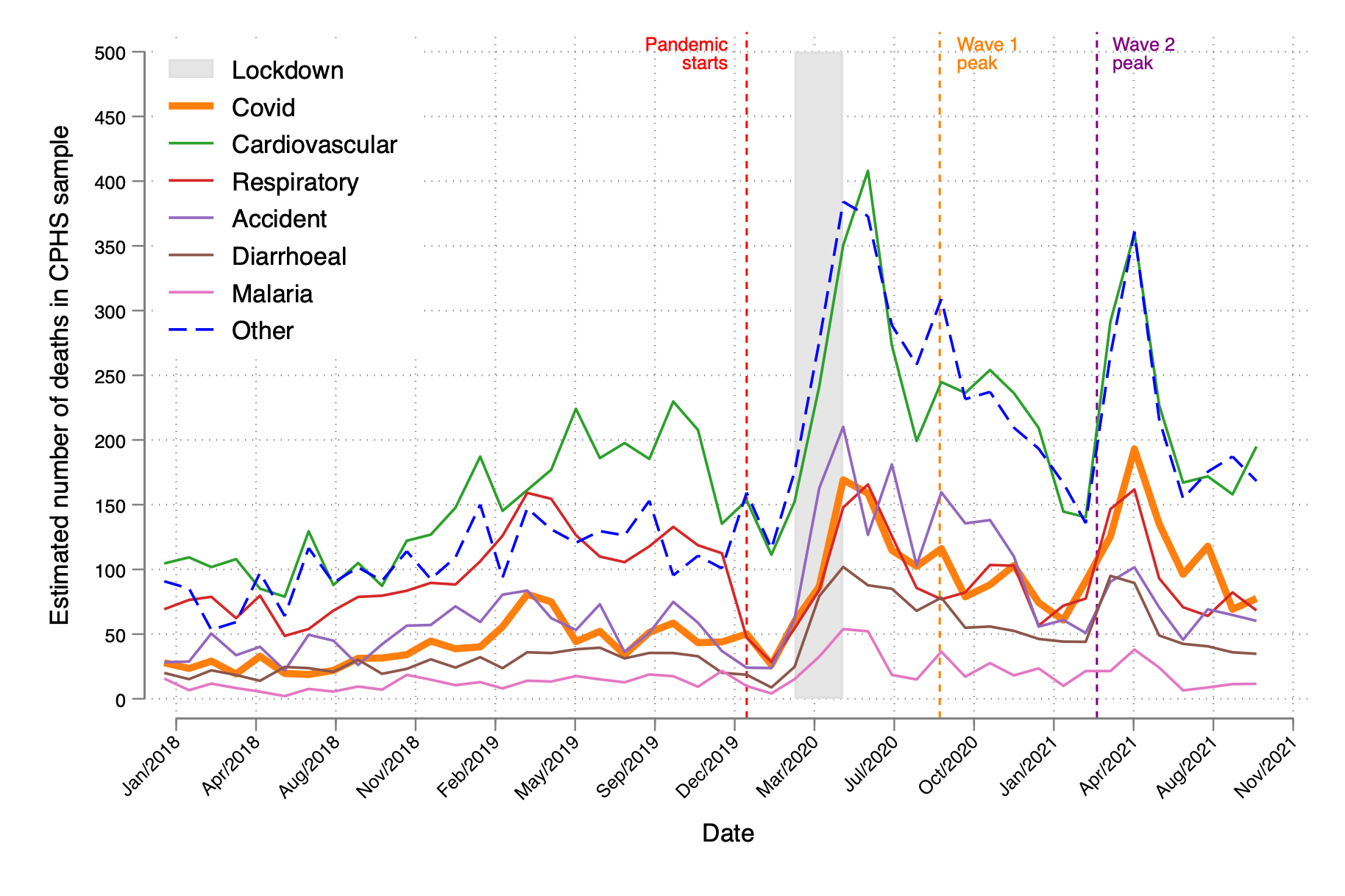


##### B: Death rate


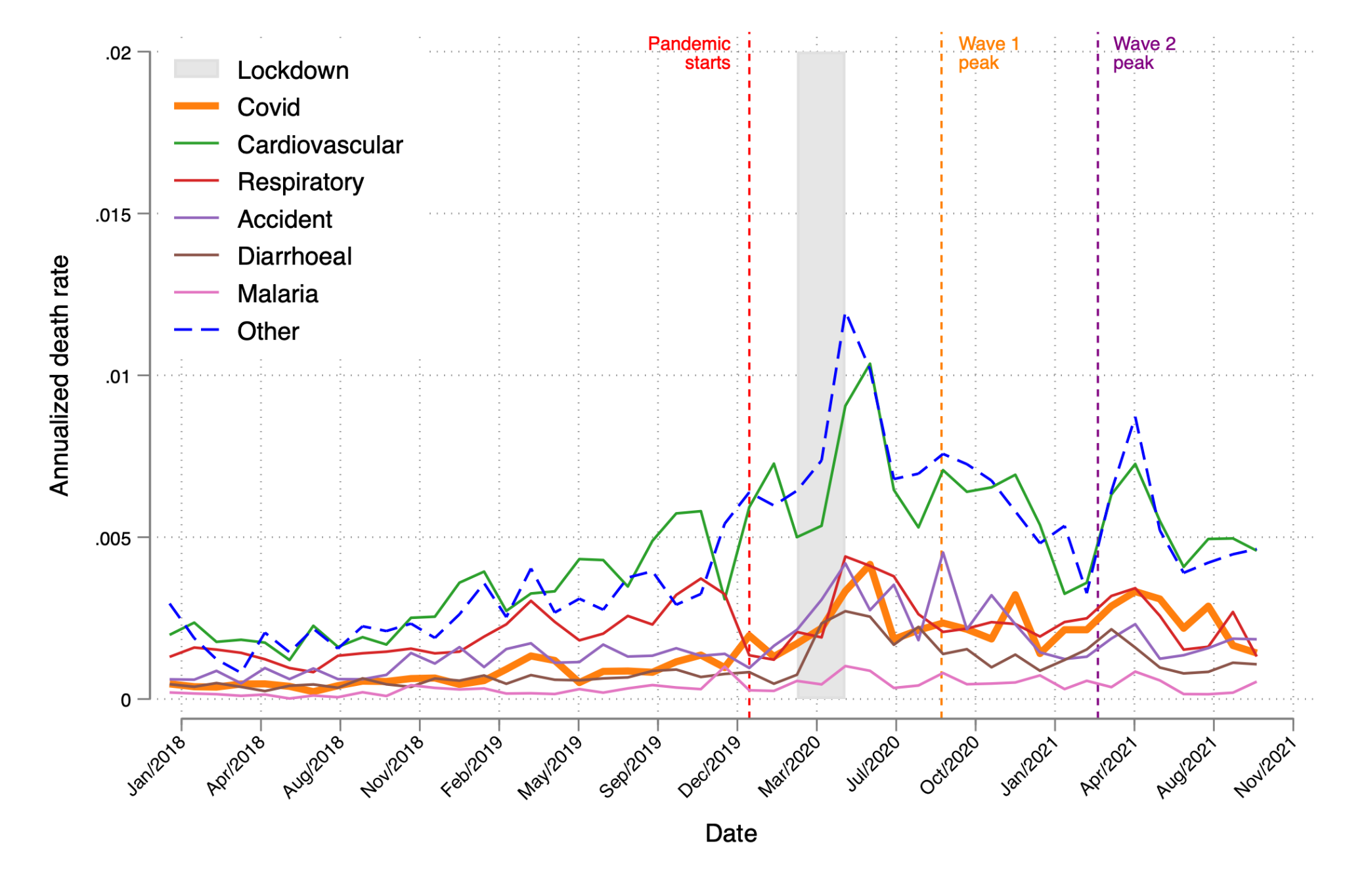


*Notes. Plot shows counts of deaths in the CPHS. These are equal to deaths recorded in the VA survey in the month of death, adjusted for non-response to the VA and the under-inclusivity of the CPHS. Date of death is defined here as the midpoint between the date a death is reported to the CPHS and the date that the decedent’s household last completed a CPHS survey.*

# References

1 Vyas, M. Survey Design and Sample. (Center for Monitoring Indian Economy, 2020).
