# Supplementary material for: The mortality burden from COVID in low-income settings: evidence from verbal autopsies in India
Source: BMC Public Health. 2026 Jan 22;26:1567. doi: 10.1186/s12889-026-26215-9 (PMC13188735; doi:10.1186/s12889-026-26215-9)
Supplement: Supplementary file 2 — Supplementary Material 2. [file 12889_2026_26215_MOESM2_ESM.pdf]

CGHR Study Adult e-VA Instrument
Questionnaire for the death of a person aged 18 years and above

Instructions for use of the tool
Questions to be read to the respondent are in bold.
[Questions that are NOT to be read to respondent are in brackets.]
Hints to the interviewer are in italic text below relevant questions. These are only hints for the interviewer and are NOT to be read to respondents.

ID	Question	Answer(s)	Skip To	Programming Notes
Before beginning the survey, ensure that the respondent is an adult.<br>**Hello, my name is** [surveyor's name] **and I am working for CMIE. We are studying ways to improve the health status of the community. We very much appreciate your participation in this survey. As part of the survey, we invite you to provide information on any deaths that happened in your household. We will ask questions related to the illness of the deceased and some other questions. This will help us understand the patterns of deaths, and will help establish how to improve health in your city/village.**				
**1005**	**What is your** [the respondent's] **MEM_ID?**	1. [Household MEM_ID list]<br>2. Someone else	→ 1006	Select- only one
**1005a**	[Please enter an identifier for the person (name, ID, etc.)]			text
**1006**	[Choose the respondent's sex.]	1. Male<br>2. Female<br>3. Transgender		Select- only one
**1007**	**How old are you in completed years?**<br>*Record the respondent's age as of her/his last birthday. Record '99' if don't know the age.*	Years __ __		[18:99]
**1016**	**What is the highest grade you have completed?**<br>*<1 year: '0', grade if only up to school level: 1-12, college/university attended but not graduated: '13', graduated: '14', never attended: '98', not known: '99'*	Grade __ __		[0:14,98:99]
**13015**	**What is your marital status?**	1. Married<br>2. Married, but Gauna not performed<br>3. Widowed<br>4. Divorced<br>5. Separated<br>6. Deserted<br>7. Never married<br>8. Engaged<br>9. Doesn't know		Select- only one
**I would like to tell you more about the study and obtain your consent.** [Read the consent form to the family.]				
**1017**	[Did the respondent give consent?]	1. Yes<br>2. No	→ 2003	

**1017a**	[Why did the respondent, in their own words, not want to do the interview?]		→ End the Interview	text,maxlength[100]
**I would now like to ask you some detailed questions about any adult deaths that have occurred in the house since January 1, 2019.**				Display ID, sex, and age (with units) of deceased from enumeration database before the following questions
**2003**	**What was the sex of the deceased?**	1. Male<br>2. Female		Select- only one
**2004**	**When was the deceased born?**	1. Day __ __<br>2. Month __ __<br>3. Year __ __ __ __		1- [1:31]<br>2- [1:12]<br>3- [2019-age-5%:2021-age+5%]
**2005**	**When did (s)he die?**	1. Day __ __<br>2. Month __ __<br>3. Year __ __ __ __		1- [1:31]<br>2- [1:12]<br>3- [2019:2021]
**2006**	**What is your** [the respondent's] **relationship to the deceased?**	1. Parent<br>2. Child<br>3. Brother/sister<br>3. Other family member<br>4. Friend<br>7. Another relationship<br>9. None of the above		Select- only one
**2011**	**Did you** [the respondent] **live with the deceased in the period leading to her/his death?**	1. Yes<br>2. No		
**2009**	**Where did the deceased die?**	1. Hospital<br>2. Other health facility<br>3. Home<br>4. On route to hospital or facility<br>5. Other<br>9. Doesn't know		Select- only one
**2019**	**What is the highest grade they completed?**<br>*<1 year: '0', grade if only up to school level: 1-12, college/university attended but not graduated: '13', graduated: '14', never attended: '98', not known: '99'*	Grade __ __		[0:14,98:99]

**13012**	**What was her/his marital status?**	1. Married<br>2. Married, but Gauna not performed<br>3. Widowed<br>4. Divorced<br>5. Separated<br>6. Deserted<br>7. Never married<br>8. Engaged<br>9. Doesn't know		Select- only one
**3001**	**What do you** [the respondent] **think the deceased died of?**			text
**6004**	**For how long was (s)he ill before death?**<br>*Less than 1 day = '0'.*	1. Days __ __<br>2. Months __ __<br>- Doesn't know		Select<br>1- [0:30]<br>2- [1:12]
**6005**	**Did (s)he die suddenly?**<br>*Suddenly means died unexpectedly within 24 hours of being in regular health*	1. Yes<br>2. No<br>9. Doesn't know		
**A004**	**Did (s)he have a test that confirmed an infection?**	1. Yes<br>2. No<br>9. Doesn't know	 → 6006<br>→ 6006	
**A005**	**What pathogen(s) was/were detected?**	1. SARS-CoV-2 (COVID-19)<br>2. Influenza<br>3. Malaria<br>4. Typhoid<br>5. Other<br>9. Doesn't know		multiselect
**6006**	**Was there any diagnosis by a health professional of the following?**<br>*Enter more than one if applicable.*<br>*Remind the respondent that we are asking for the diagnosis assessed by a doctor, health worker, or other health professional PRIOR to the final illness.*	1. Heart disease<br>2. High blood pressure<br>3. Diabetes<br>4. Stroke<br>5. Chronic lung disease<br>6. Asthma<br>7. Cancer<br>8. Kidney disease<br>9. Liver disease<br>10. Depression<br>11. Tuberculosis<br>12. HIV/AIDS<br>13. Other (specify) ________________________<br>14. None of the above		multiselect

**3002**	[Select what you believe the respondent reported as the cause of death. If they do not know, then enter "Does not know".]	1. Pneumonia<br>2. TB<br>3. Chronic respiratory disease<br>4. Diarrhoea<br>5. Infections / typhoid / viral fever / jaundice / fever<br>6. Injury/accident<br>7. Measles<br>8. Meningitis (brain fever)<br>9. Malaria<br>10. Nutritional/malnutrition<br>11. Kidney problem / endocrine disease<br>12. Liver problem<br>13. Cancer<br>14. HIV/STI<br>15. Heart disease<br>16. Stroke<br>17. Digestive disease<br>18. Maternal conditions<br>19. Other or unknown cause		Based on the respondent's stated COD captured here, apply symptom sequence (attached separately) to determine the order of the questions in Sections 5-7<br><br>In the eVA, options will be suggested as the surveyor types, which are then automatically classified into one of these categories.
**5001**	**Did (s)he suffer from any injury or accident that led to her/his death?**	1. Yes<br>2. No<br>9. Doesn't know	 → Next symptom<br>→ Next symptom	

**5002**	**What was the nature of the injury or accident?**	1. Road traffic accident<br>2. Non-road transport accident<br>3. Drowning<br>4. Fall<br>5. Injury by falling object / blunt force<br>6. Pesticide poisoning<br>7. Other poisoning<br>8. Hanging<br>10. Electrocution<br>11. Burns/fire<br>12. Injured by a firearm<br>13. Bite or sting<br>14. Stabbed/cut/pierced<br>15. Strangled<br>16. Flood<br>17. Earthquake<br>18. Other (specify) ________________________<br>9. Doesn't know	→ 5007<br><br><br><br><br><br><br><br><br><br><br>→ 5009<br><br><br>→ Next symptom<br>→ Next symptom	Select- only one
**5003**	**Was (s)he subject to violence (suicide, homicide, abuse)?**	1. Yes<br>2. No<br>9. Doesn't know		
**5004**	**Was the injury accidental?**	1. Yes<br>2. No<br>9. Doesn't know	→ Next symptom	
**5005**	**Was the injury self-inflicted?**	1. Yes<br>2. No<br>9. Doesn't know	→ Next symptom	
**5006**	**Was the injury intentionally inflicted by someone else?**	1. Yes<br>2. No<br>9. Doesn't know	→ All: Next symptom	
**5007**	**What was her/his role in the road traffic accident?**	1. Pedestrian<br>2. Driver or passenger in car or light vehicle<br>3. Driver or passenger in bus or heavy vehicle<br>4. Driver or passenger on a motorcycle<br>5. Driver or passenger on a pedal cycle<br>6. Other<br>9. Doesn't know		Select- only one

**5008**	**What was the counterpart that was hit during the road traffic accident?**	1. Pedestrian<br>2. Stationary object<br>3. Car or light vehicle<br>4. Bus or heavy vehicle<br>5. Motorcycle<br>6. Pedal cycle<br>7. Other<br>9. Doesn't know	→ All: Next symptom	Select- only one
**5009**	**What was the animal/insect?**	1. Dog<br>2. Snake<br>3. Insect or scorpion<br>4. Other<br>9. Doesn't know		Select- only one
**7001**	**Did (s)he have a fever?**	1. Yes<br>2. No<br>9. Doesn't know	 → 7007<br>→ 7007	If yes, reminders about fever will appear in the narrative section
**7003**	**How long did the fever last?**<br>*Less than 1 day = '0'.*	1. Days __ __<br>2. Months __ __<br>- Doesn't know		Select<br>1- [0:30]<br>2- [1:60]
**7004**	**Did the fever continue until death?**	1. Yes<br>2. No<br>9. Doesn't know		
**7005**	**How severe was the fever?**	1. Mild<br>2. Moderate<br>3. Severe<br>9. Doesn't know		Select- only one
**7006**	**What was the pattern of the fever?**	1. Continuous<br>2. On and off<br>3. Only at night<br>9. Doesn't know		Select- only one
**7007**	**Did (s)he have night sweats?**	1. Yes<br>2. No<br>9. Doesn't know		
**A008**	**Did (s)he experience a disturbance in her/his sense of taste or smell?**	1. Yes<br>2. No<br>9. Doesn't know		
**A009**	**Did (s)he feel fatigue or malaise?**	1. Yes<br>2. No<br>9. Doesn't know		

**7057**	**Did (s)he have a severe headache?**	1. Yes<br>2. No<br>9. Doesn't know		
**A010**	**Did (s)he have muscle aches (myalgia)?**	1. Yes<br>2. No<br>9. Doesn't know		
**7058**	**Did (s)he have a stiff or painful neck during the illness that led to death?**	1. Yes<br>2. No<br>9. Doesn't know	 → Next symptom<br>→ Next symptom	
**7059**	**Did (s)he have a stiff neck during illness that led to death?**	1. Yes<br>2. No<br>9. Doesn't know	 → 7061<br>→ 7061	
**7060**	**How long before death did (s)he have stiff neck?**<br>*Less than 1 day = '0'.*	1. Days __ __<br>2. Months __ __<br>- Doesn't know		Select<br>1- [0:30]<br>2- [1:60]
**7061**	**Did (s)he have a painful neck during the illness that led to death?**	1. Yes<br>2. No<br>9. Doesn't know	 → Next symptom<br>→ Next symptom	
**7062**	**How long before death did (s)he have a painful neck?**<br>*Less than 1 day = '0'.*	1. Days __ __<br>2. Months __ __<br>- Doesn't know		Select<br>1- [0:30]<br>2- [1:60]
**7008**	**Did (s)he have a cough?**	1. Yes<br>2. No<br>9. Doesn't know	 → A012<br>→ A012	If yes, reminders about cough will appear in the narrative section
**7009**	**For how long did (s)he have a cough?**<br>*Less than 1 day = '0'.*	1. Days __ __<br>2. Months __ __<br>- Doesn't know		Select<br>1- [0:30]<br>2- [1:60]
**7010**	**Was the cough productive, with sputum?**	1. Yes<br>2. No<br>9. Doesn't know		Select- only one
**7011**	**Was the cough very severe?**	1. Yes<br>2. No<br>9. Doesn't know		
**7012**	**Did (s)he cough up blood?**	1. Yes<br>2. No<br>9. Doesn't know		
**A012**	**Did (s)he have a sore throat?**	1. Yes<br>2. No<br>9. Doesn't know		

**7014**	**Did (s)he have a breathing problem?** (e.g. difficulty breathing, fast breathing, breathlessness, wheezing)	1. Yes<br>2. No<br>9. Doesn't know	 → Next symptom<br>→ Next symptom	If yes, reminders about breathing problems will appear in the narrative section
**7015**	**Did (s)he have any difficulty breathing?**	1. Yes<br>2. No<br>9. Doesn't know	 → 7019<br>→ 7019	
**7017**	**For how long did the difficult breathing last?**<br>*Less than 1 day = '0'.*	1. Days __ __<br>2. Months __ __<br>3. Years __ __<br>- Doesn't know		Select<br>1- [0:30]<br>2- [1:60]<br>3- <Age
**7018**	**Was the difficulty continuous or on and off?**	1. Continuous<br>2. On and off<br>9. Doesn't know		Select- only one
**7019**	**During the illness that led to death, did (s)he have fast breathing?**	1. Yes<br>2. No<br>9. Doesn't know	 → 7022<br>→ 7022	
**7021**	**How long did the fast breathing last?**<br>*Less than 1 day = '0'.*	1. Days __ __<br>2. Months __ __<br>- Doesn't know		Select<br>1- [0:30]<br>2- [1:60]
**7022**	**Did (s)he have breathlessness?**	1. Yes<br>2. No<br>9. Doesn't know	 → 7029<br>→ 7029	
**7024**	**How long did (s)he have breathlessness?**<br>*Less than 1 day = '0'.*	1. Days __ __<br>2. Months __ __<br>- Doesn't know		Select<br>1- [0:30]<br>2- [1:60]
**7025**	**Was (s)he unable to carry out daily routines due to breathlessness?**	1. Yes<br>2. No<br>9. Doesn't know		
**7026**	**Was (s)he breathless while lying flat?**	1. Yes<br>2. No<br>9. Doesn't know		
**7029**	**During the illness that led to death did (s)he have wheezing?**	1. Yes<br>2. No<br>9. Doesn't know		
**7030**	**Did (s)he have chest pain?**	1. Yes<br>2. No<br>9. Doesn't know	 → Next symptom<br>→ Next symptom	If yes, reminders about chest pain will appear in the narrative section

**7031**	**Was the chest pain severe?**	1. Yes<br>2. No<br>9. Doesn't know		
**7032**	**How many days before death did (s)he have chest pain?**<br>*Less than 1 day = '0'.*	Days __ __<br>- Doesn't know		[0:30]
**7033**	**How long did the chest pain last?**<br>*Less than 1 minute = '0'.*	1. Minutes __ __<br>2. Hours __ __<br>3. Days __ __<br>- Doesn't know		Select- only one<br>1- [0:59]<br>2- [1:23]<br>3- [1:30],<Days Q7032
**7034**	**Did (s)he have more frequent loose or liquid stools than usual?**<br>*Ask the respondent about his/her understanding of what diarrhoea is (having more frequent loose or liquid stools than usual); if unclear or wrong, explain to the respondent what diarrhoea is.*	1. Yes<br>2. No<br>9. Doesn't know	 → 7040<br>→ 7040	If yes, reminders about loose or liquid stools will appear in the narrative section
**7035**	**How long did (s)he have frequent loose or liquid stools?**<br>*Less than 1 day = '0'.*	1. Days __ __<br>2. Months __ __<br>- Doesn't know		Select<br>1- [0:30]<br>2- [1:60]
**7040**	**At any time during the final illness was there blood in the stools?**	1. Yes<br>2. No<br>9. Doesn't know	 → Next symptom<br>→ Next symptom	
**7041**	**Was there blood in the stool up until death?**	1. Yes<br>2. No<br>9. Doesn't know		
**7042**	**Did (s)he vomit?**	1. Yes<br>2. No<br>9. Doesn't know	 → Next symptom<br>→ Next symptom	If yes, reminders about vomiting will appear in the narrative section
**7043**	**To clarify: Did (s)he vomit in the week preceding the death?**	1. Yes<br>2. No<br>9. Doesn't know	 → Next symptom<br>→ Next symptom	Skip changes to 7045 if 1 was selected in Q7042
**7044**	**How long before death did (s)he vomit?**<br>*Less than 1 day = '0'.*	1. Days __ __<br>2. Months __ __<br>- Doesn't know		Select<br>1- [0:30]<br>2- [1:60]
**7045**	**Was there blood in the vomit?**	1. Yes<br>2. No<br>9. Doesn't know		
**7046**	**Was the vomit black?**	1. Yes<br>2. No<br>9. Doesn't know		

**7047**	**Did (s)he have any belly (abdominal) problem?**<br>*Explain to the respondent that problems could be pain, protruding abdomen or a mass.*	1. Yes<br>2. No<br>9. Doesn't know	 → 7114<br>→ 7114	If yes, reminders about stomach problems will appear in the narrative section
**7048**	**Did (s)he have belly (abdominal) pain?**	1. Yes<br>2. No<br>9. Doesn't know	 → 7052<br>→ 7052	
**7049**	**Was the belly (abdominal) pain severe?**	1. Yes<br>2. No<br>9. Doesn't know		
**7050**	**For how long did (s)he have belly (abdominal) pain?**<br>*Less than 1 day = '0'.*	1. Hours __ __<br>2. Days __ __<br>3. Months __ __<br>- Doesn't know		Select<br>1- [0:23]<br>2- [1:30]<br>3- [1:60]
**7051**	**Was the pain in the upper or lower belly (abdomen)?**	1. Upper abdomen<br>2. Lower abdomen<br>3. Upper and lower abdomen<br>9. Doesn't know		Select- only one
**7052**	**Did (s)he have a more than usually protruding belly (abdomen)?**	1. Yes<br>2. No<br>9. Doesn't know	 → 7055<br>→ 7055	
**7053**	**For how long before death did (s)he have a more than usually protruding belly (abdomen)?**<br>*Less than 1 day = '0'.*	1. Days __ __<br>2. Months __ __<br>- Doesn't know		Select<br>1- [0:30]<br>2- [1:60]
**7054**	**How rapidly did (s)he develop the protruding belly (abdomen)?**	1. Rapidly<br>2. Slowly<br>9. Doesn't know		Select- only one
**7055**	**Did (s)he have any mass in the belly (abdomen)?**	1. Yes<br>2. No<br>9. Doesn't know	 → 7114<br>→ 7114	
**7056**	**For how long did (s)he have a mass in the belly (abdomen)?**<br>*Less than 1 day = '0'.*	1. Days __ __<br>2. Months __ __<br>- Doesn't know		Select<br>1- [0:30]<br>2- [1:60]
**7114**	**Did (s)he have difficulty swallowing?**	1. Yes<br>2. No<br>9. Doesn't know	 → 7117<br>→ 7117	
**7115**	**For how long before death did (s)he have difficulty swallowing?**<br>*Less than 1 day = '0'.*	1. Days __ __<br>2. Months __ __<br>- Doesn't know		Select<br>1- [0:30]<br>2- [1:60]

**7116**	**Was the difficulty with swallowing with solids, liquids, or both?**	1. Solids<br>2. Liquids<br>3. Both<br>9. Doesn't know		Select- only one
**7117**	**Did (s)he have pain upon swallowing?**	1. Yes<br>2. No<br>9. Doesn't know		
**7093**	**During the illness that led to death, did (s)he bleed from anywhere?**	1. Yes<br>2. No<br>9. Doesn't know	 → Next symptom<br>→ Next symptom	
**7094**	**Did (s)he bleed from the nose, mouth or anus?**	1. Yes<br>2. No<br>9. Doesn't know		
**7065**	**Was (s)he unconscious during the illness that led to death?**	1. Yes<br>2. No<br>9. Doesn't know	 → 7063<br>→ 7063	If yes, reminders about unconsciousness will appear in the narrative section
**7066**	**Was (s)he unconscious for more than 24 hours before death?**	1. Yes<br>2. No<br>9. Doesn't know		
**7068**	**Did the unconsciousness start suddenly, quickly (at least within a single day)?**	1. Yes<br>2. No<br>9. Doesn't know		
**7069**	**Did the unconsciousness continue until death?**	1. Yes<br>2. No<br>9. Doesn't know		
**7063**	**Did (s)he have mental confusion?**	1. Yes<br>2. No<br>9. Doesn't know	 → Next symptom<br>→ Next symptom	
**7064**	**How long did (s)he have mental confusion?**<br>*Less than 1 day = '0'.*	1. Days __ __<br>2. Months __ __<br>- Doesn't know		Select<br>1- [0:30]<br>2- [1:60]
**7070**	**Did (s)he have convulsions?**	1. Yes<br>2. No<br>9. Doesn't know	 → Next symptom<br>→ Next symptom	If yes, reminders about convulsions will appear in the narrative section
**7072**	**For how many minutes did the convulsions last?**<br>*Less than 1 minute = '0'. Use 1 hour=60 minutes to determine the number of minutes.*	Minutes __ __<br>- Doesn't know		[0:60]

**7073**	**Did (s)he become unconscious immediately after the convulsion?**	1. Yes<br>2. No<br>9. Doesn't know		
**7074**	**Did (s)he have any urine problems?**<br>*Explain to the respondent that urinary problems refer to urinating a lot or not at all, and blood in the urine.*	1. Yes<br>2. No<br>9. Doesn't know	 → Next symptom<br>→ Next symptom	If yes, reminders about urinary problems will appear in the narrative section
**7075**	**Did (s)he go to urinate more often than usual?**	1. Yes<br>2. No<br>9. Doesn't know		
**7076**	**During the final illness did (s)he ever pass blood in the urine?**	1. Yes<br>2. No<br>9. Doesn't know		
**7077**	**Did (s)he stop urinating?**	1. Yes<br>2. No<br>9. Doesn't know		
**7078**	**Did (s)he have any skin problems, ulcers, or sores?**	1. Yes<br>2. No<br>9. Doesn't know	 → A015<br>→ A015	If yes, reminders about skin problems will appear in the narrative section
**7079**	**Did (s)he have sores or ulcers anywhere on the body?**	1. Yes<br>2. No<br>9. Doesn't know		
**7080**	**Did (s)he have sores?**	1. Yes<br>2. No<br>9. Doesn't know	 → 7082<br>→ 7082	
**7081**	**Did the sores have clear fluid or pus?**	1. Yes<br>2. No<br>9. Doesn't know		
**7082**	**Did (s)he have an ulcer (pit) on the foot?**	1. Yes<br>2. No<br>9. Doesn't know	 → 7085<br>→ 7085	
**7083**	**Did the ulcer on the foot ooze pus?**	1. Yes<br>2. No<br>9. Doesn't know	 → 7085<br>→ 7085	
**7084**	**How long did the ulcer on the foot ooze pus?**<br>*Less than 1 day = '0'.*	1. Days __ __<br>2. Months __ __<br>- Doesn't know		Select<br>1- [0:30]<br>2- [1:60]

**7085**	**During the illness that led to death, did (s)he have any skin rash?**	1. Yes<br>2. No<br>9. Doesn't know	 → 7089<br>→ 7089	
**7086**	**For how many days did (s)he have the skin rash?**<br>*Less than 1 day = '0'.*	Days __ __<br>- Doesn't know		[0:30]
**7087**	**Where was the rash?**	1. Face<br>2. Trunk or abdomen<br>3. Extremities<br>4. Everywhere<br>9. Doesn't know		Select- only one
**7088**	**Did (s)he have measles rash (use local term)?**	1. Yes<br>2. No<br>9. Doesn't know		
**7089**	**Did (s)he ever have shingles or herpes zoster?**	1. Yes<br>2. No<br>9. Doesn't know		
**7090**	**During the illness that led to death, did her/his skin flake off in patches?**	1. Yes<br>2. No<br>9. Doesn't know		
**A015**	**Did the deceased report any itchy red, pink, or purple rash or swelling on their toes in the weeks prior to death?**	1. Yes<br>2. No<br>9. Doesn't know		
**7095**	**Did (s)he have noticeable weight loss?**	1. Yes<br>2. No<br>9. Doesn't know		If yes, reminders about weight loss will appear in the narrative section
**7096**	**Was (s)he severely thin or wasted?**	1. Yes<br>2. No<br>9. Doesn't know		
**7097**	**During the illness that led to death, did (s)he have a whitish rash inside the mouth or on the tongue?**	1. Yes<br>2. No<br>9. Doesn't know		
**7098**	**Did (s)he have stiffness of the whole body or inability to open the mouth?**	1. Yes<br>2. No<br>9. Doesn't know		
**7120**	**Did her/his hair change in colour to a reddish or yellowish colour?**	1. Yes<br>2. No<br>9. Doesn't know		

7121	**Did (s)he look pale (thinning/lack of blood) or have pale palms, eyes or nail beds?**<br>*Long term deficiency of the blood results in a pale, whitish appearance of the lips, tongue, and eye sac. Sometimes it is referred to as thinning or lack of blood, or pallor.*	1. Yes<br>2. No<br>9. Doesn't know		
7123	**Did (s)he drink a lot more water than usual?**	1. Yes<br>2. No<br>9. Doesn't know		
7099	**Did (s)he have oedema/swelling?**	1. Yes<br>2. No<br>9. Doesn't know	 → Next symptom<br>→ Next symptom	If yes, reminders about oedema/swelling will appear in the narrative section
7100	**Did (s)he have puffiness of the face?**	1. Yes<br>2. No<br>9. Doesn't know	 → 7102<br>→ 7102	
7101	**How long did (s)he have puffiness of the face?**<br>*Less than 1 day = '0'.*	1. Days __ __<br>2. Months __ __<br>- Doesn't know		Select<br>1- [0:30]<br>2- [1:60]
7102	**During the illness that led to death, did (s)he have swollen legs or feet?**	1. Yes<br>2. No<br>9. Doesn't know	 → 7105<br>→ 7105	
7103	**How long did the swelling last?**<br>*Less than 1 day = '0'.*	1. Days __ __<br>2. Months __ __<br>- Doesn't know		Select<br>1- [0:30]<br>2- [1:60]
7104	**Did (s)he have both feet swollen?**	1. Yes<br>2. No<br>9. Doesn't know		
7105	**Did (s)he have general puffiness all over his/her body?**	1. Yes<br>2. No<br>9. Doesn't know		
7106	**Did (s)he have any lumps?**	1. Yes<br>2. No<br>9. Doesn't know	 → Next symptom<br>→ Next symptom	If yes, reminders about lumps will appear in the narrative section
7107	**Did (s)he have any lumps or lesions in the mouth?**	1. Yes<br>2. No<br>9. Doesn't know		
7108	**Did (s)he have any lumps on the neck?**	1. Yes<br>2. No<br>9. Doesn't know		

**7109**	**Did (s)he have any lumps on the armpit?**	1. Yes<br>2. No<br>9. Doesn’t know		
**7110**	**Did (s)he have any lumps on the groin?**	1. Yes<br>2. No<br>9. Doesn’t know		
**7111**	**Was (s)he in any way paralysed?**	1. Yes<br>2. No<br>9. Doesn’t know	 → Next symptom<br>→ Next symptom	If yes, reminders about paralysis will appear in the narrative section
**7112**	**Did (s)he have paralysis of only one side of the body?**	1. Yes<br>2. No<br>9. Doesn’t know		
**7113**	**Which were the limbs or body parts paralysed?**<br>*Enter more than one if applicable.*	1. Right side<br>2. Left side<br>3. Lower part of body<br>4. Upper part of body<br>5. One leg only<br>6. One arm only<br>7. Whole body<br>8. Other (specify) ___________________________		multiselect
**7118**	**Did (s)he have yellow discoloration of the eyes?**	1. Yes<br>2. No<br>9. Doesn’t know	 → Next symptom<br>→ Next symptom	If yes, reminders about jaundice will appear in the narrative section
**7119**	**For how long did (s)he have the yellow discoloration?**<br>*Less than 1 day = ‘0’.*	1. Days __ __<br>2. Months __ __<br>- Doesn’t know		Select<br>1- [0:30]<br>2- [1:60]
**SIGNS AND SYMPTOMS ASSOCIATED WITH PREGNANCY AND WOMEN**				Section enabled only for female deaths
**7144**	**Did she have any swelling or lump in the breast?**	1. Yes<br>2. No<br>9. Doesn’t know		
**7145**	**Did she have any ulcers (pits) in the breast?**	1. Yes<br>2. No<br>9. Doesn’t know		
**7146**	**Did she ever have a period or menstruate?**	1. Yes<br>2. No<br>9. Doesn’t know	 → 7154<br>→ 7154	

**7147**	**When she had her period, did she have vaginal bleeding in between menstrual periods?**	1. Yes<br>2. No<br>9. Doesn't know	 → 7149<br>→ 7149	
**7148**	**Was the bleeding excessive?**	1. Yes<br>2. No<br>9. Doesn't know		
**7149**	**Was there excessive vaginal bleeding in the week prior to death?**	1. Yes<br>2. No<br>9. Doesn't know		
**7150**	**Did her menstrual period stop naturally because of menopause or removal of the uterus?**	1. Yes<br>2. No<br>9. Doesn't know	→ 7153	Enabled only for deaths aged ≥40 years
**7151**	**At the time of death was her period overdue?**	1. Yes<br>2. No<br>9. Doesn't know	 → 7154<br>→ 7154	Enabled only for deaths aged 15-49 years; otherwise, skip to 7154
**7152**	**For how many weeks had her period been overdue?**	Weeks __ __<br>- Doesn't know	→ All: 7154	[0:8]<br>Enabled only for deaths aged 15-49 years
**7153**	**Did she have vaginal bleeding after cessation of menstruation?**	1. Yes<br>2. No<br>9. Doesn't know	→ All: 7160	Enabled only for female deaths aged ≥40 years
**7154**	**Did she have a sharp pain in her belly (abdomen) shortly before death?**	1. Yes<br>2. No<br>9. Doesn't know		
**7155**	**Was she pregnant at the time of death?**	1. Yes<br>2. No<br>9. Doesn't know	→ 7159	
**7156**	**Did she die within 6 weeks of delivery, abortion or miscarriage?**	1. Yes<br>2. No<br>9. Doesn't know	→ 7161	
**7157**	**Did this woman die more than 6 weeks after being pregnant or delivering a baby?**	1. Yes<br>2. No<br>9. Doesn't know	 → 7160<br>→ 7160	
**7158**	**Was this a woman who died less than 1 year after being pregnant or delivering a baby?**	1. Yes<br>2. No<br>9. Doesn't know	→ 7161<br>→ 7160<br>→ 7160	
**7159**	**For how many months was she pregnant?**	Months __ __<br>- Doesn't know	→ All: 7161	[1:10]

**7160**	**Please confirm: when she died, she was NEITHER pregnant NOR had delivered, had an abortion, or miscarried within 12 months of when she died – is that right?**	1. Yes (she was not pregnant, and she did not recently deliver, have abortion, or miscarry)<br>2. No (she was pregnant or she recently delivered, had an abortion, or miscarried)<br>9. Doesn't know	→ Next symptom	
**7161**	**Did she ever have a period or menstruate?**	1. Yes<br>2. No<br>9. Doesn't know	 → 7169<br>→ 7169	
**7162**	**When she had her period, did she have vaginal bleeding in between menstrual periods?**	1. Yes<br>2. No<br>9. Doesn't know	 → 7164<br>→ 7164	
**7163**	**Was the bleeding excessive?**	1. Yes<br>2. No<br>9. Doesn't know		
**7164**	**Was there excessive vaginal bleeding in the week prior to death?**	1. Yes<br>2. No<br>9. Doesn't know		
**7165**	**Did her menstrual period stop naturally because of menopause or removal of the uterus?**	1. Yes<br>2. No<br>9. Doesn't know	→ 7168	Enabled only for deaths aged ≥40 years
**7166**	**At the time of death was her period overdue?**	1. Yes<br>2. No<br>9. Doesn't know	 → 7169<br>→ 7169	Enabled only for deaths aged 15-49 years; otherwise, skip to 7169
**7167**	**For how many weeks had her period been overdue?**	Weeks __ __<br>- Doesn't know	→ All: 7169	[0:8]<br>Enabled only for deaths aged 15-49 years
**7168**	**Did she have vaginal bleeding after cessation of menstruation?**	1. Yes<br>2. No<br>9. Doesn't know	→ All: 7175	Enabled only for female deaths aged ≥40 years
**7169**	**Did she have a sharp pain in her belly (abdomen) shortly before death?**	1. Yes<br>2. No<br>9. Doesn't know		
**7170**	**Was she pregnant at the time of death?**	1. Yes<br>2. No<br>9. Doesn't know	→ 7174	
**7171**	**Did she die within 6 weeks of delivery, abortion or miscarriage?**	1. Yes<br>2. No<br>9. Doesn't know	→ 7176	

**7172**	**Did this woman die more than 6 weeks after being pregnant or delivering a baby?**	1. Yes<br>2. No<br>9. Doesn't know	 → 7175<br>→ 7175	
**7173**	**Was this a woman who died less than 1 year after being pregnant or delivering a baby?**	1. Yes<br>2. No<br>9. Doesn't know	→ 7176<br>→ 7175<br>→ 7175	
**7174**	**For how many months was she pregnant?**	Months __ __<br>- Doesn't know	→ All: 7176	[1:10]
**7175**	**Please confirm: when she died, she was NEITHER pregnant NOR had delivered, had an abortion, or miscarried within 12 months of when she died – is that right?**	1. Yes (she was not pregnant, and she did not recently deliver, have abortion, or miscarry)<br>2. No (she was pregnant or she recently delivered, had an abortion, or miscarried)<br>9. Doesn't know	→ Next symptom<br><br><br><br>→ Next symptom	
**7176**	**Did she die during labour or delivery?**	1. Yes<br>2. No<br>9. Doesn't know	→ 7180	
**7177**	**Did she die after delivering a baby?**	1. Yes<br>2. No<br>9. Doesn't know	 → 7180<br>→ 7180	
**7178**	**Did she die within 24 hours after delivery?**	1. Yes<br>2. No<br>9. Doesn't know	→ 7180	
**7179**	**Did she die within 6 weeks of childbirth?**	1. Yes<br>2. No<br>9. Doesn't know		
**7180**	**Did she give birth to a live baby (within 6 weeks of her death)?**	1. Yes<br>2. No<br>9. Doesn't know		
**7181**	**Did she die during or after a multiple pregnancy?**	1. Yes<br>2. No<br>9. Doesn't know	→ All: 7183	Skip is implemented if 1 is chosen in Q7176 or Q7178, or 2 or 9 is chosen in Q7180
**7182**	**Was she breastfeeding the child in the days before death?**	1. Yes<br>2. No<br>9. Doesn't know		
**7183**	**How many births, including stillbirths, did she/the mother have before this baby?**	Births __ __<br>- Doesn't know		[0:15]

**7184**	**Had she had any previous Caesarean section?**	1. Yes<br>2. No<br>9. Doesn't know		
**7185**	**During pregnancy, did she suffer from high blood pressure?**	1. Yes<br>2. No<br>9. Doesn't know		
**7186**	**Did she have foul smelling vaginal discharge during pregnancy or after delivery?**	1. Yes<br>2. No<br>9. Doesn't know		
**7187**	**During the last 3 months of pregnancy, did she suffer from convulsions?**	1. Yes<br>2. No<br>9. Doesn't know		
**7188**	**During the last 3 months of pregnancy did she suffer from blurred vision?**	1. Yes<br>2. No<br>9. Doesn't know		
**7189**	**Did bleeding occur while she was pregnant?**	1. Yes<br>2. No<br>9. Doesn't know	 → 7192<br>→ 7192	
**7190**	**Was there vaginal bleeding during the first 6 months of pregnancy?**	1. Yes<br>2. No<br>9. Doesn't know		
**7191**	**Was there vaginal bleeding during the last 3 months of pregnancy but before labour started?**	1. Yes<br>2. No<br>9. Doesn't know		
**7192**	**Did she have excessive bleeding during labour or delivery?**	1. Yes<br>2. No<br>9. Doesn't know		
**7193**	**Did she have excessive bleeding after delivery or abortion?**	1. Yes<br>2. No<br>9. Doesn't know		
**7194**	**Was the placenta completely delivered?**	1. Yes<br>2. No<br>9. Doesn't know		
**7195**	**Did she deliver or try to deliver an abnormally positioned baby?**	1. Yes<br>2. No<br>9. Doesn't know		
**7196**	**For how many hours was she in labour?**<br>*Less than 1 hour = '0'.*	Hours __ __<br>- Doesn't know		[0:72]

**7197**	**Did she attempt to terminate the pregnancy?**	1. Yes<br>2. No<br>9. Doesn't know	→ All: 7201	Skip is implemented if 1 was chosen for Q7180
**7198**	**Did she recently have a pregnancy that ended in an abortion (spontaneous or induced)?**	1. Yes<br>2. No<br>9. Doesn't know	 → 7201<br>→ 7201	
**7199**	**Did she die during an abortion?**	1. Yes<br>2. No<br>9. Doesn't know		
**7200**	**Did she die within 6 weeks of having an abortion?**	1. Yes<br>2. No<br>9. Doesn't know		
**7201**	**Where did she give birth?**	1. Hospital<br>2. Other health facility<br>3. Home<br>4. On route to hospital or facility<br>5. Other<br>9. Doesn't know		Select- only one
**7202**	**Did she receive professional assistance during the delivery?**<br>*Explain to the respondent what is meant by professional assistance: delivery attended by a medical professional (doctor, nurse, or midwife).*	1. Yes<br>2. No<br>9. Doesn't know		
**7203**	**Who delivered the baby?**	1. Doctor<br>2. Midwife<br>3. Nurse<br>4. Relative<br>5. Self (the mother)<br>6. Traditional birth attendant<br>7. Other<br>9. Doesn't know		Select- only one
**7204**	**Did she have an operation to remove her uterus shortly before death?**	1. Yes<br>2. No<br>9. Doesn't know		
**7205**	**Was the delivery normal vaginal, without forceps or vacuum?**	1. Yes<br>2. No<br>9. Doesn't know	→ 7208	

**7206**	**Was the delivery vaginal, with forceps or vacuum?**	1. Yes<br>2. No<br>9. Doesn't know	→ 7208	
**7207**	**Was the delivery a Caesarean section?**	1. Yes<br>2. No<br>9. Doesn't know		
**7208**	**Was the baby born more than one month early?**	1. Yes<br>2. No<br>9. Doesn't know		
**I would like to ask some questions concerning habits and the mood that the deceased had; some of these questions may not appear to be directly related to her/his death. Please bear with me and answer all the questions. They will help us to get a clear picture of all possible risk factors.**				
**8004**	**Did (s)he use any form of tobacco within the last 5 years?**	1. Yes<br>2. No<br>9. Doesn't know	 → 8001<br>→ 8001	
**8005**	**What was the method of tobacco use?**<br>*Enter more than one if applicable.*	1. Cigarette<br>2. Bidi<br>3. Chew or apply tobacco<br>9. Doesn't know	 <br><br>→ 8001	multiselect
**8006**	**How many cigarettes were consumed per day?**	Number __ __	→ 8001	[0:99]<br>Skip is implemented if 2 or 3 was not chosen in Q8005
**8008**	**How many bidis were smoked per day?**	Number __ __	→ 8001	[0:99]<br>Skip is implemented if 3 was not chosen in Q8005
**8007**	**How many times per day did they chew/apply tobacco?**	Number __ __		[0:99]
**8001**	**Did (s)he normally drink alcohol at least once a week during most weeks in the last 5 years?**<br>*Use local term for alcohol.*	1. Yes<br>2. No<br>9. Doesn't know	 → 2017<br>→ 2017	
**8002**	**What was the average number of days per week (s)he drank?**	Number __ __<br>- Doesn't know		[1:7]
**8003**	**What type of alcohol was most commonly consumed?**	1. Local liquor (toddy)<br>2. Foreign liquor<br>3. Beer<br>4. Other<br>9. Doesn't know		Select- only one

**2017**	**Did the deceased have in the several years PRIOR to becoming ill, any of the following for at least 2 continuous weeks?**<br>**Feeling much sadder, or more depressed than usual, OR**<br>**Loss of interest in most things like hobbies or activities that usually gave you pleasure, OR**<br>**Feeling sad or hopeless so that you lost your appetite, OR**<br>**Feeling worthless and useless, so that life was very difficult and there was no way out**	1. Yes<br>2. No<br>9. Doesn't know	 → 11001<br>→ 11001	Select- only one
**2018**	**How old were they at the LAST such episode?**	Years __ __		Maximum is deceased age
**KEY SYMPTOMS**				
**11001**	**You said that (s)he had the following symptoms.**<br>[List of positive symptoms]<br>**Which one occurred first? Second?** [Continue until all symptoms have been put in order.]<br>*If '1' was previously selected for only one symptom, do not ask this question. If respondent does not know the order, enter the symptoms in the order in which they appear above.*	1.<br>2.<br>3.<br>4.<br>5.<br>6.		Enabled only if '1. Yes' was selected for more than one symptom<br>ALL IMPORTANT POSITIVE SYMPTOMS APPEAR, and the program requests the surveyor to put them in chronological order - FOLLOW EXACT SAME approach as in current e-VA
	**Thank you for the information. You mentioned that the deceased had some problems before death. Can you please tell me about any medical treatment that they received for these problems?**<br>*USE THIS SPACE to take notes on details of medical treatment that the deceased received during the illness that led to death, as well as any additional probing of positive symptoms during the narrative. For paper forms, refer to the reminders (attached separately) for additional information to probe for each positive symptom.*<br>____________________<br>____________________<br>____________________<br>____________________<br>____________________<br>____________________			The format for the notes section is the same as in the current e-VA app.<br><br>On left-hand side, summary of answers from probing of all positive symptoms appears in chronological order.<br><br>Recording begins at the start of this section.

**11002**	[Narrative] **Thank you for your information. Now can you please tell me in your own words about the events that led to the death?**<br>*Using the notes, read out the organised history to the respondent and get her/his confirmation before writing it below.*<br>**Excuse me for a few minutes while I write out what you have told me.**<br>*Please put in chronological order the key symptoms, any treatments received, and probing questions. Remember your goal is NOT to arrive at a diagnosis (that will be done by the central doctors) but the KEY symptoms and the KEY sickness history in chronological order.*<br>____			Minimum of 300 characters to be entered in the narrative text box<br><br>Recording ends after completion of this question.
**I would like to ask some questions about the treatments that the deceased received and health services that were accessed.**				
**9001**	**Did (s)he receive treatment for the illness that led to death?**	1. Yes<br>2. No<br>9. Doesn't know	 → A031<br>→ A031	
**9003**	**Did (s)he receive (or need) intravenous fluids (drip) treatment?**	1. Yes<br>2. No<br>9. Doesn't know		
**9004**	**Did (s)he receive (or need) a blood transfusion?**	1. Yes<br>2. No<br>9. Doesn't know		
**9005**	**Did (s)he receive (or need) treatment/food through a tube passed through the nose?**	1. Yes<br>2. No<br>9. Doesn't know		

**9006**	**Did (s)he receive (or need) injectable antibiotics?**	1. Yes<br>2. No<br>9. Doesn't know		
**9007**	**Did (s)he receive (or need) antiretroviral therapy (ART)?**	1. Yes<br>2. No<br>9. Doesn't know		
**A020**	**Did (s)he ever require supplemental oxygen through a mask or through the nose?**	1. Yes<br>2. No<br>9. Doesn't know		
**A021**	**Did (s)he ever require intubation (breathing machine)?**	Select one:<br>1. Yes<br>2. No<br>9. Doesn't know		Select- only one
**9008**	**Did (s)he receive (or need) an operation for the illness?**	1. Yes<br>2. No<br>9. Doesn't know	 → 9010<br>→ 9010	
**9009**	**Did (s)he have the operation within 1 month before death?**	1. Yes<br>2. No<br>9. Doesn't know		
**9010**	**Was (s)he discharged from hospital very ill?**	1. Yes<br>2. No<br>9. Doesn't know		
**A031**	**Had (s)he been vaccinated against COVID?**<br>*Answer 'Yes' if (s)he received at least one dose of a COVID vaccine.*	1. Yes<br>2. No<br>9. Doesn't know	 → 9015<br>→ 9015	
**A032**	**How may doses of the COVID vaccine had (s)he received?**	1. One dose<br>2. Two doses<br>3. More than two doses<br>9. Doesn't know		Select- only one
**A033**	**For their first dose which vaccine did (s)he receive?**	1. Indian CoVaxin<br>2. Moderna or Pfizer mRNA vaccine<br>3. CoviShield/AstraZeneca vaccine<br>4. Sputnik 5 vaccine<br>5. Other<br>9. Don't know	→ 9015<br>→ 9015<br>→ 9015<br>→ 9015<br><br>→ 9015	Select- only one<br><br>Skip changes to A034 if 2 or 3 was selected in QA032
**A033a**	**Specify the vaccine.**			text,maxlength[30]

**A034**	**For their second dose which vaccine did (s)he receive?**	1. Indian CoVaxin<br>2. Moderna or Pfizer mRNA vaccine<br>3. CoviShield/AstraZeneca vaccine<br>4. Sputnik 5 vaccine<br>5. Other<br>9. Don't know	→ 9015<br>→ 9015<br>→ 9015<br>→ 9015<br><br>→ 9015	Select- only one
**A034a**	**Specify the vaccine for the second dose.**			text,maxlength[30]
**9015**	**Was care sought outside the home while (s)he had this illness?**	1. Yes<br>2. No<br>9. Doesn't know	 → 9018<br>→ 9018	
**9016**	**Where or from whom did you seek this care?**<br>*Enter more than one if applicable.*	1. Government Hospital<br>2. Government Health centre or clinic<br>3. Private Hospital<br>4. Ayush (Ayurveda, Unani, etc.)<br>5. Relative, friend (outside household)<br>6. Other<br>9. Doesn't know	 <br><br>→ 9018<br>→ 9018<br>→ 9018<br>→ 9018	multiselect<br><br>Skips are implemented if none of 1, 2, and 3 are chosen
**A022**	**Did (s)he ever get admitted to an intensive care unit?**	1. Yes<br>2. No<br>9. Doesn't know		
**9018**	**Did a health care worker tell you the cause of death?**	1. Yes<br>2. No<br>9. Doesn't know	 → 9025<br>→ 9025	
**9019**	**What did the health care worker say?**			text
**9025**	**In the final days before death, did (s)he travel to a hospital or health facility?**	1. Yes<br>2. No<br>9. Doesn't know	 → 9030<br>→ 9030	
**9026**	**Did (s)he use an ambulance to get to the hospital or health facility?**	1. Yes<br>2. No<br>9. Doesn't know		
**9027**	**Were there any problems during admission to the hospital or health facility?**	1. Yes<br>2. No<br>9. Doesn't know		
**9028**	**Were there any problems with the way (s)he was treated (medical treatment, procedures, interpersonal attitudes, respect, dignity) in the hospital or health facility?**	1. Yes<br>2. No<br>9. Doesn't know		

**9029**	**Were there any problems getting medications or diagnostic tests in the hospital or health facility?**	1. Yes<br>2. No<br>9. Doesn't know		
**9030**	**Does it take more than 2 hours to get to the nearest hospital or health facility from the deceased's household?**	1. Yes<br>2. No<br>9. Doesn't know		
**9031**	**In the final days before death, were there any doubts about whether medical care was needed?**	1. Yes<br>2. No<br>9. Doesn't know		
**9032**	**In the final days before death, was traditional medicine used?**	1. Yes<br>2. No<br>9. Doesn't know		
**9033**	**In the final days before death, did anyone use a telephone or cell phone to call for help?**	1. Yes<br>2. No<br>9. Doesn't know		
**9034**	**Over the course of illness, did the total costs of care and treatment interfere with other routine household expenses?**	1. Yes<br>2. No<br>9. Doesn't know		
**9035**	**Did the household have to borrow money or sell any household items to pay for treatment during the illness?**	1. Yes<br>2. No<br>9. Doesn't know		
**9036**	**Was the death registered in the local government system (municipal or civil registration)?**	1. Yes<br>2. No<br>9. Doesn't know		
**9037**	**Did you receive a doctor's death certificate with the cause of death?**	1. Yes<br>2. No<br>9. Doesn't know	 → A023<br>→ A023	
**9038**	**Can I see the death certificate?**	1. Yes<br>2. No	 → A023	
**9038a**	[Enter the cause of death.]			text
**9038b**	[Enter the date on the certificate.]	1. Day __ __<br>2. Month __ __<br>3. Year __ __ __ __		1- [1:31]<br>2- [1:12]<br>3- [2019:2022]
**Sources of exposure**				
**A023**	**In the two weeks before (s)he became sick, did the deceased travel anywhere outside their home district?**	1. Yes<br>2. No<br>9. Doesn't know	 → A025<br>→ A025	

**A024**	**Where did (s)he travel?**			text
**A025**	**In the two weeks before (s)he became sick, did (s)he have close contact with a confirmed or probable case of SARS-CoV-2 infection / COVID-19?**<br>*Close contact is defined as: healthcare-associated exposure, including providing direct care for COVID patients; working together in close proximity or sharing the same classroom environment with a COVID patient; traveling together with a COVID patient in any kind of conveyance; or living in the same household as a COVID patient.*	1. Yes<br>2. No<br>9. Doesn't know		
**Let me thank you for your patience in answering these questions. I know it is difficult to talk about a loved family member's death, but your information will help prevent such deaths in the future. Before I conclude, a few short questions about YOUR health:**				
**1018**	**How is your current general health status?**	1. Excellent<br>2. Good<br>3. Fair<br>4. Poor		
**1019**	**Did you use any form of tobacco within the last 5 years?**	1. Yes<br>2. No<br>9. Doesn't know	 → 1031<br>→ 1031	
**1020**	**What was the method of tobacco use?**<br>*Enter more than one if applicable.*	1. Cigarette<br>2. Bidi<br>3. Chew or apply tobacco<br>9. Doesn't know	 <br><br>→ 1030	multiselect
**1021**	**How many cigarettes smoked per day?**	Number __ __	→ 1030	[0:99]<br>Skip is implemented if 2 or 3 was not chosen in Q1020
**1027**	**How many bidis smoked per day?**	Number __ __	→ 1030	[0:99]<br>Skip is implemented if 3 was not chosen in Q1020
**1022**	**How many times did you chew/apply tobacco per day?**	Number __ __		[0:99]

**1031**	**Have you had a diagnosis by a health professional of the following?**<br>*Enter more than one if applicable.*<br>*Remind the respondent that we are asking for the diagnosis assessed by a doctor, health worker, or other health professional during the final illness.*	1. Heart disease<br>2. High blood pressure<br>3. Diabetes<br>4. Stroke<br>5. Chronic lung disease<br>6. Asthma<br>7. Cancer<br>8. Kidney disease<br>9. Liver disease<br>10. Depression<br>11. Tuberculosis<br>13. Other (specify) ________________________<br>14. None of the above		multiselect
**1028**	**Have you had in the last several years when you were not otherwise sick, any of the following for at least 2 continuous weeks?**<br>**Feeling much sadder, or more depressed than usual, OR**<br>**Loss of interest in most things like hobbies or activities that usually gave you pleasure, OR**<br>**Feeling sad or hopeless so that you lost your appetite, OR**<br>**Feeling worthless and useless, so that life was very difficult and there was no way out**	1. Yes<br>2. No<br>9. Doesn't know	 → 1031<br>→ 1031	Select- only one
**1029**	**How old were you at the LAST such episode?**	Completed years __ __		Maximum is respondent age
**Before I conclude, two short questions for you:**				
**12098**	**Would you say the interview was:**	1. Too long<br>2. Too short<br>3. About right length<br>9. No comment		Select- only one
**12099**	**Do you have any suggestions on how we can do better?**			text,maxlength[200]
NOTE: THE FOLLOWING QUESTIONS ARE FOR AUTO-POPULATION AND ARE NOT TO BE ASKED TO RESPONDENTS.				
**13004**	[Name of VA interviewer]	Text		
**13005**	[Time at start of interview]	[hh: ___ ___mm ___ ___]		
**13006**	[Date of interview]	[dd/mm/yyyy]		
**13021**	[Time at end of interview]	[hh: ___ ___mm ___ ___]		
