# Supplementary material for: The mortality burden from COVID in low-income settings: evidence from verbal autopsies in India
Source: BMC Public Health. 2026 Jan 22;26:1567. doi: 10.1186/s12889-026-26215-9 (PMC13188735; doi:10.1186/s12889-026-26215-9)
Supplement: Supplementary file 3 — Supplementary Material 3. [file 12889_2026_26215_MOESM3_ESM.pdf]

CGHR Study Child e-VA Instrument
Questionnaire for the death of a child aged 28 days to 11 years

<u>Instructions for use of the tool</u>
Questions to be read to the respondent are in bold.
[Questions that are NOT to be read to respondent are in brackets.]
Hints to the interviewer are in italic text below relevant questions. These are only hints for the interviewer and are NOT to be read to respondents.

ID	Question	Answer(s)	Skip To	Programming Notes
Before beginning the survey, ensure that the respondent is an adult.<br>**Hello, my name is** [surveyor's name] **and I am working for CMIE. We are studying ways to improve the health status of the community. We very much appreciate your participation in this survey. As part of the survey, we invite you to provide information on any deaths that happened in your household. We will ask questions related to the illness of the deceased and some other questions. This will help us understand the patterns of deaths, and will help establish how to improve health in your city/village.**				
**1005**	**What is your** [the respondent's] **MEM_ID?**	1. [Household MEM_ID list]<br>2. Someone else	→ 1006	Select- only one
**1005a**	[Please enter an identifier for the person (name, ID, etc.)]			text
**1006**	[Choose the respondent's sex.]	1. Male<br>2. Female<br>3. Transgender		Select- only one
**1007**	**How old are you in completed years?**<br>*Record the respondent's age as of her/his last birthday. Record '99' if don't know the age.*	Years __ __		[18:99]
**1016**	**What is the highest grade you have completed?**<br>*<1 year: '0', grade if only up to school level: 1-12, college/university attended but not graduated: '13', graduated: '14', never attended: '98', not known: '99'*	Grade __ __		[0:14,98:99]
**13015**	**What is your marital status?**	1. Married<br>2. Married, but Gauna not performed<br>3. Widowed<br>4. Divorced<br>5. Separated<br>6. Deserted<br>7. Never married<br>8. Engaged<br>9. Doesn't know		Select- only one
**I would like to tell you more about the study and obtain your consent.** [Read the consent form to the family.]				
**1017**	[Did the respondent give consent?]	1. Yes<br>2. No	→ 2003	

**1017a**	[Why did the respondent, in their own words, not want to do the interview?]		→ End the Interview	text,maxlength[100]
**I would now like to ask you some detailed questions about any child deaths that have occurred in the house since January 1, 2019.**				Display name, sex, and age (with units) of deceased from enumeration database before the following questions
**2003**	**What was the sex of the deceased?**	1. Male<br>2. Female		Select- only one
**2004**	**When was the deceased born?**	1. Day __ __<br>2. Month __ __<br>3. Year __ __ __ __		1- [1:31]<br>2- [1:12]<br>3- [2019-age-5%:2021-age+5%]
**2005**	**When did (s)he die?**	1. Day __ __<br>2. Month __ __<br>3. Year __ __ __ __		1- [1:31]<br>2- [1:12]<br>3- [2019:2021]
**2006**	**What is your/the respondent's relationship to the deceased?**	1. Parent<br>2. Brother/sister<br>3. Other family member<br>4. Friend<br>5. Another relationship<br>6. None of the above		Select- only one
**2011**	**Did you/the respondent live with the deceased in the period leading to her/his death?**	1. Yes<br>2. No		
**2009**	**Where did the deceased die?**	1. Hospital<br>2. Other health facility<br>3. Home<br>4. On route to hospital or facility<br>5. Other<br>9. Doesn't know		Select- only one
**2019**	**What is the highest grade they completed?**<br>*<1 year: '0', grade if only up to school level: 1-12, never attended: '98', not known: '99'*	Grade __ __		[0:12,98:99]
**3001**	**What do you (respondent) think the deceased died of?**<br>*Record the respondent's own exact words.*			text

**3002**	[Select what you believe the respondent reported as the cause of death. If they do not know, then enter "Does not know".]	1. Pneumonia/TB/asthma<br>2. Diarrhoea<br>3. Infections / typhoid / viral fever / jaundice / fever<br>4. Injury/accident<br>5. Measles<br>6. Meningitis (brain fever)<br>7. Malaria<br>8. Nutritional/malnutrition<br>9. Kidney problem / endocrine disease<br>10. Liver problem<br>11. Cancer<br>12. Other or unknown cause		Based on the respondent's stated COD captured here, apply symptom sequence (attached separately) to determine the order of the questions in Sections 5 & 7<br><br>In the eVA, options will be suggested as the surveyor types, which are then automatically classified into one of these categories.
**6002**	**Before the illness that led to death, was the child growing normally?**	1. Yes<br>2. No<br>9. Doesn't know		
**6004**	**For how long was (s)he ill before death?**<br>*Less than 1 day = '0'.*	1. Days __ __<br>2. Months __ __<br>- Doesn't know		Select<br>1- [0:30]<br>2- [1:12]
**6005**	**Did (s)he die suddenly?**<br>*Suddenly means died unexpectedly within 24 hours of being in regular health*	1. Yes<br>2. No<br>9. Doesn't know		
**6006**	**Was there any diagnosis by a health professional of the following?**<br>*Enter more than one if applicable.*<br>*Remind the respondent that we are asking for the diagnosis assessed by a doctor, health worker, or other health professional PRIOR to the final illness.*	1. Tuberculosis<br>2. HIV/AIDS<br>3. Recent positive test for malaria<br>3. Dengue fever<br>4. Measles<br>5. Heart disease<br>6. Diabetes<br>7. Asthma<br>8. Cancer<br>9. Kidney disease<br>10. Liver disease<br>11. Other (specify) ________________________<br>12. None of the above		multiselect
**5001**	**Did (s)he suffer from any injury or accident that led to her/his death?**	1. Yes<br>2. No<br>9. Doesn't know	 → Next symptom<br>→ Next symptom	

**5002**	**What was the nature of the injury or accident?**<br>*Hanging option is applicable only if age at death ≥10 years*	1. Road traffic accident<br>2. Non-road transport accident<br>3. Drowning<br>4. Fall<br>5. Injury by falling object / blunt force<br>6. Pesticide poisoning<br>7. Other poisoning<br>8. Hanging<br>10. Electrocution<br>11. Burns/fire<br>12. Injured by a firearm<br>13. Bite or sting<br>14. Stabbed/cut/pierced<br>15. Strangled<br>16. Flood<br>17. Earthquake<br>18. Other<br>9. Doesn't know	→ 5007<br><br><br><br><br><br><br><br><br><br><br>→ 5009<br><br><br>→ Next symptom<br>→ Next symptom	8. Hanging option enabled only for age ≥10 years
**5003**	**Was (s)he subject to violence (homicide, abuse)?**	1. Yes<br>2. No<br>9. Doesn't know		
**5004**	**Was the injury accidental?**	1. Yes<br>2. No<br>9. Doesn't know	→ Next symptom	
**5006**	**Was the injury intentionally inflicted by someone else?**	1. Yes<br>2. No<br>9. Doesn't know	→ All: Next symptom	
**5007**	**What was her/his role in the road traffic accident?**	1. Pedestrian<br>2. Driver or passenger in car or light vehicle<br>3. Driver or passenger in bus or heavy vehicle<br>4. Driver or passenger on a motorcycle<br>5. Driver or passenger on a pedal cycle<br>6. Other<br>9. Doesn't know		Select- only one

**5008**	**What was the counterpart that was hit during the road traffic accident?**	1. Pedestrian<br>2. Stationary object<br>3. Car or light vehicle<br>4. Bus or heavy vehicle<br>5. Motorcycle<br>6. Pedal cycle<br>7. Other<br>9. Doesn't know	→ All: Next symptom	Select- only one
**5009**	**What was the animal/insect?**	1. Dog<br>2. Snake<br>3. Insect or scorpion<br>4. Other<br>9. Doesn't know		Select- only one
**7001**	**Did (s)he have a fever?**	1. Yes<br>2. No<br>9. Doesn't know	 → 7007<br>→ 7007	
**7003**	**For how long did the fever last?**<br>*Less than 1 day = '0'.*	1. Days __ __<br>2. Months __ __<br>- Doesn't know		Days,Months=<Age at Death<br>Select<br>1- [0:30]<br>2- [1:60]
**7004**	**Did the fever continue until death?**	1. Yes<br>2. No<br>9. Doesn't know		
**7005**	**How severe was the fever?**	1. Mild<br>2. Moderate<br>3. Severe<br>9. Doesn't know		Select- only one
**7006**	**What was the pattern of the fever?**	1. Continuous<br>2. On and off<br>3. Only at night<br>9. Doesn't know		Select- only one
**7007**	**Did (s)he have night sweats?**	1. Yes<br>2. No<br>9. Doesn't know		
**7057**	**Did (s)he have a severe headache?**	1. Yes<br>2. No<br>9. Doesn't know		

**7059**	**Did (s)he have a stiff neck during illness that led to death?**	1. Yes<br>2. No<br>9. Doesn't know	 → 7061<br>→ 7061	
**7060**	**How long before death did (s)he have stiff neck?**<br>*Less than 1 day = '0'.*	1. Days __ __<br>2. Months __ __<br>- Doesn't know		Days,Months=<Age at Death<br>Select<br>1- [0:30]<br>2- [1:60]
**7061**	**Did (s)he have a painful neck during the illness that led to death?**	1. Yes<br>2. No<br>9. Doesn't know	 → Next symptom<br>→ Next symptom	
**7062**	**How long before death did (s)he have a painful neck?**<br>*Less than 1 day = '0'.*	1. Days __ __<br>2. Months __ __<br>- Doesn't know		Days,Months=<Age at Death<br>Select<br>1- [0:30]<br>2- [1:60]
**7008**	**Did (s)he have a cough?**	1. Yes<br>2. No<br>9. Doesn't know	 → 7014<br>→ 7014	
**7009**	**For how long did (s)he have a cough?**<br>*Less than 1 day = '0'.*	1. Days __ __<br>2. Months __ __<br>- Doesn't know		Days,Months=<Age at Death<br>Select<br>1- [0:30]<br>2- [1:60]
**7010**	**Was the cough productive, with sputum?**	1. Yes<br>2. No<br>9. Doesn't know		
**7011**	**Was the cough very severe?**	1. Yes<br>2. No<br>9. Doesn't know		
**7012**	**Did (s)he cough up blood?**	1. Yes<br>2. No<br>9. Doesn't know		
**7013**	**Did (s)he make a whooping sound when coughing?**	1. Yes<br>2. No<br>9. Doesn't know		
**7015**	**Did (s)he have any difficulty breathing?**	1. Yes<br>2. No<br>9. Doesn't know	 → 7019<br>→ 7019	

**7017**	**For how long did the difficult breathing last?**<br>*Less than 1 day = '0'.*	1. Days __ __<br>2. Months __ __<br>3. Years __ __<br>- Doesn't know		Days,Months,Years=<Age at Death<br>Select<br>1- [0:30]<br>2- [1:11]
**7018**	**Was the difficulty continuous or on and off?**	1. Continuous<br>2. On and off<br>9. Doesn't know		Select- only one
**7019**	**During the illness that led to death, did (s)he have fast breathing?**	1. Yes<br>2. No<br>9. Doesn't know	 → 7022<br>→ 7022	
**7021**	**How long did the fast breathing last?**<br>*Less than 1 day = '0'.*	1. Days __ __<br>2. Months __ __<br>- Doesn't know		Select<br>1- [0:30]<br>2- [1:60]
**7022**	**Did (s)he have breathlessness?**	1. Yes<br>2. No<br>9. Doesn't know	 → 7027<br>→ 7027	
**7024**	**How long did (s)he have breathlessness?**<br>*Less than 1 day = '0'.*	1. Days __ __<br>2. Months __ __<br>- Doesn't know		Days,Months=<Age at Death<br>Select<br>1- [0:30]<br>2- [1:60]
**7027**	**Did you see the lower chest wall/ribs being pulled in as the child breathed in?**<br>*Show photos/video (if available).*	1. Yes<br>2. No<br>9. Doesn't know		
**7028**	**During the illness that led to death did his/her breathing sound like any of the following:**	1. Stridor<br>2. Grunting<br>3. Wheezing<br>4. None of the above<br>9. Doesn't know		Select- only one
**7030**	**Did (s)he have chest pain?**	1. Yes<br>2. No<br>9. Doesn't know	 → Next symptom<br>→ Next symptom	
**7032**	**How many days before death did (s)he have chest pain?**<br>*Less than 1 day = '0'.*	Days __ __<br>- Doesn't know		[0:30]

**7034**	**Did (s)he have more frequent loose or liquid stools than usual?**<br>*Ask the respondent about his/her understanding of what is diarrhoea (having more frequent loose or liquid stools than usual); if unclear or wrong, explain to the respondent what diarrhoea is.*	1. Yes<br>2. No<br>9. Doesn't know	 → 7040<br>→ 7040	
**7035**	**How long did (s)he have frequent loose or liquid stools?**<br>*Less than 1 day = '0'.*	1. Days __ __<br>2. Months __ __<br>- Doesn't know		Select<br>1- [0:30]<br>2- [1:60]
**7036**	**How many stools did the child have on the day that loose or liquid stools were most frequent?**	No. of stools: __ __<br>- Doesn't know		[1:25]
**7038**	**How long before death did the frequent loose or liquid stools start?**<br>*Less than 1 day = '0'.*	1. Days __ __<br>2. Months __ __<br>- Doesn't know		Select<br>1- [0:30]<br>2- [1:60]
**7039**	**Did the frequent loose or liquid stools continue until death?**	1. Yes<br>2. No<br>9. Doesn't know		
**7040**	**At any time during the final illness was there blood in the stools?**	1. Yes<br>2. No<br>9. Doesn't know	 → 7122<br>→ 7122	
**7041**	**Was there blood in the stool up until death?**	1. Yes<br>2. No<br>9. Doesn't know		
**7122**	**Did (s)he have sunken eyes?**	1. Yes<br>2. No<br>9. Doesn't know		
**7123**	**Did (s)he drink a lot more water than usual?**	1. Yes<br>2. No<br>9. Doesn't know		
**7042**	**Did (s)he vomit?**	1. Yes<br>2. No<br>9. Doesn't know	 → Next symptom<br>→ Next symptom	
**7043**	**To clarify: Did (s)he vomit in the week preceding death?**	1. Yes<br>2. No<br>9. Doesn't know	 → Next symptom<br>→ Next symptom	No skip if 1 was selected in Q7042
**7045**	**Was there blood in the vomit?**	1. Yes<br>2. No<br>9. Doesn't know		

**7046**	**Was the vomit black?**	1. Yes<br>2. No<br>9. Doesn't know		
**7047**	**Did (s)he have any belly (abdominal) problem?**<br>*Explain to the respondent that problems could be pain, protruding abdomen or a mass.*	1. Yes<br>2. No<br>9. Doesn't know	 → 7114<br>→ 7114	
**7048**	**Did (s)he have belly (abdominal) pain?**	1. Yes<br>2. No<br>9. Doesn't know	 → 7052<br>→ 7052	
**7049**	**Was the belly (abdominal) pain severe?**	1. Yes<br>2. No<br>9. Doesn't know		
**7050**	**For how long did (s)he have belly (abdominal) pain?**<br>*Less than 1 day = '0'.*	1. Hours __ __<br>2. Days __ __<br>3. Months __ __<br>- Doesn't know		Days,Months=<Age at Death<br>Select<br>1- [0:24]<br>2- [1:30]<br>3- [1:60]
**7051**	**Was the pain in the upper or lower belly (abdomen)?**	1. Upper abdomen<br>2. Lower abdomen<br>3. Upper and lower abdomen<br>9. Doesn't know		Select- only one
**7052**	**Did (s)he have a more than usually protruding belly (abdomen)?**	1. Yes<br>2. No<br>9. Doesn't know	 → 7055<br>→ 7055	
**7053**	**For how long before death did (s)he have a more than usually protruding belly (abdomen)?**<br>*Less than 1 day = '0'.*	1. Days __ __<br>2. Months __ __<br>- Doesn't know		Days,Months=<Age at Death<br>Select<br>1- [0:30]<br>2- [1:60]
**7054**	**How rapidly did (s)he develop the protruding belly (abdomen)?**	1. Rapidly<br>2. Slowly<br>9. Doesn't know		
**7055**	**Did (s)he have any mass in the belly (abdomen)?**	1. Yes<br>2. No<br>9. Doesn't know	 → 7114<br>→ 7114	
**7056**	**For how long did (s)he have a mass in the belly (abdomen)?**<br>*Less than 1 day = '0'.*	1. Days __ __<br>2. Months __ __<br>- Doesn't know		Days,Months=<Age at Death<br>Select<br>1- [0:30]<br>2- [1:60]

**7114**	**Did (s)he have difficulty swallowing?**	1. Yes<br>2. No<br>9. Doesn't know	 → 7117<br>→ 7117	
**7115**	**For how long before death did (s)he have difficulty swallowing?**<br>*Less than 1 day = '0'.*	Days __ __<br>- Doesn't know		[0:30]
**7116**	**Was the difficulty with swallowing with solids, liquids, or both?**	1. Solids<br>2. Liquids<br>3. Both<br>9. Doesn't know		Select- only one
**7117**	**Did (s)he have pain upon swallowing?**	1. Yes<br>2. No<br>9. Doesn't know		
**7065**	**Was (s)he unconscious during the illness that led to death?**	1. Yes<br>2. No<br>9. Doesn't know	 → Next symptom<br>→ Next symptom	
**7066**	**Was (s)he unconscious for more than 24 hours before death?**	1. Yes<br>2. No<br>9. Doesn't know	→ 7068<br><br>→ 7068	
**7067**	**How long before death did unconsciousness start?**<br>*Less than 1 hour = '0'.*	1. Hours __ __<br>2. Days __ __<br>- Doesn't know		Select<br>1- [0:24]<br>2- [1:99]
**7068**	**Did the unconsciousness start suddenly, quickly (at least within a single day)?**	1. Yes<br>2. No<br>9. Doesn't know		
**7069**	**Did the unconsciousness continue until death?**	1. Yes<br>2. No<br>9. Doesn't know		
**7070**	**Did (s)he have convulsions?**	1. Yes<br>2. No<br>9. Doesn't know	 → Next symptom<br>→ Next symptom	
**7071**	**Did (s)he experience any generalized convulsions or fits during the illness that led to death?**	1. Yes<br>2. No<br>9. Doesn't know		
**7072**	**For how many minutes did the convulsions last?**<br>*Less than 1 minute = '0'. Use 1 hour=60 minutes to determine the number of minutes.*	Minutes __ __<br>- Doesn't know		[0:60]

**7073**	**Did (s)he become unconscious immediately after the convulsion?**	1. Yes<br>2. No<br>9. Doesn't know		
**7074**	**Did (s)he have any urine problems?**<br>*Explain to the respondent that urinary problems refer to urinating a lot or not at all, and blood in the urine.*	1. Yes<br>2. No<br>9. Doesn't know	 → Next symptom<br>→ Next symptom	
**7075**	**Did (s)he go to urinate more often than usual?**	1. Yes<br>2. No<br>9. Doesn't know		
**7076**	**During the final illness did (s)he ever pass blood in the urine?**	1. Yes<br>2. No<br>9. Doesn't know		
**7077**	**Did (s)he stop urinating?**	1. Yes<br>2. No<br>9. Doesn't know		
**7079**	**Did (s)he have sores or ulcers anywhere on the body?**	1. Yes<br>2. No<br>9. Doesn't know	 → 7082<br>→ 7082	
**7081**	**Did the sores have clear fluid or pus?**	1. Yes<br>2. No<br>9. Doesn't know		
**7082**	**Did (s)he have an ulcer (pit) on the foot?**	1. Yes<br>2. No<br>9. Doesn't know	 → 7085<br>→ 7085	
**7083**	**Did the ulcer on the foot ooze pus?**	1. Yes<br>2. No<br>9. Doesn't know	 → 7085<br>→ 7085	
**7084**	**How long did the ulcer on the foot ooze pus?**<br>*Less than 1 day = '0'.*	1. Days __ __<br>2. Months __ __<br>- Doesn't know		Select<br>1- [0:30]<br>2- [1:60]
**7085**	**During the illness that led to death, did (s)he have any skin rash?**	1. Yes<br>2. No<br>9. Doesn't know	 → 7090<br>→ 7090	
**7086**	**For how many days did (s)he have the skin rash?**<br>*Less than 1 day = '0'.*	Days __ __<br>- Doesn't know		[0:30]

**7087**	**Where was the rash?**	1. Face<br>2. Trunk or abdomen<br>3. Extremities<br>4. Everywhere<br>9. Doesn't know		Multi-select
**7088**	**Did (s)he have measles rash** (use local term)**?**	1. Yes<br>2. No<br>9. Doesn't know		
**7090**	**During the illness that led to death, did her/his skin flake off in patches?**	1. Yes<br>2. No<br>9. Doesn't know		
**7091**	**During the illness that led to death, did (s)he have areas of the skin that turned black?**	1. Yes<br>2. No<br>9. Doesn't know		
**7092**	**During the illness that led to death, did (s)he have areas of the skin with redness and swelling?**	1. Yes<br>2. No<br>9. Doesn't know		
**7093**	**During the illness that led to death, did (s)he bleed from anywhere?**	1. Yes<br>2. No<br>9. Doesn't know		
**7095**	**Did (s)he have noticeable weight loss?**	1. Yes<br>2. No<br>9. Doesn't know		
**7096**	**Was (s)he severely thin or wasted?**	1. Yes<br>2. No<br>9. Doesn't know		
**7097**	**During the illness that led to death, did (s)he have a whitish rash inside the mouth or on the tongue?**	1. Yes<br>2. No<br>9. Doesn't know		
**7098**	**Did (s)he have stiffness of the whole body or was unable to open the mouth?**	1. Yes<br>2. No<br>9. Doesn't know		
**7120**	**Did her/his hair change in colour to a reddish or yellowish colour?**	1. Yes<br>2. No<br>9. Doesn't know		

**7121**	**Did (s)he look pale (thinning/lack of blood) or have pale palms, eyes or nail beds?**<br>*Long term deficiency of the blood results in a pale, whitish appearance of the lips, tongue, and eye sac. Sometimes it is referred to as thinning or lack of blood, or pallor.*	1. Yes<br>2. No<br>9. Doesn't know		
**7100**	**Did (s)he have puffiness of the face?**	1. Yes<br>2. No<br>9. Doesn't know	 → 7102<br>→ 7102	
**7101**	**How long did (s)he have puffiness of the face?**<br>*Less than 1 day = '0'.*	1. Days __ __<br>2. Months __ __<br>- Doesn't know		Days,Months=<Age at Death<br>Select<br>1- [0:30]<br>2- [1:60]
**7102**	**During the illness that led to death, did (s)he have swollen legs or feet?**	1. Yes<br>2. No<br>9. Doesn't know	 → 7105<br>→ 7105	
**7103**	**How long did the swelling last?**<br>*Less than 1 day = '0'.*	1. Days __ __<br>2. Months __ __<br>- Doesn't know		Days,Months=<Age at Death<br>Select<br>1- [0:30]<br>2- [1:60]
**7104**	**Did (s)he have both feet swollen?**	1. Yes<br>2. No<br>9. Doesn't know		
**7105**	**Did (s)he have general puffiness all over his/her body?**	1. Yes<br>2. No<br>9. Doesn't know		
**7106**	**Did (s)he have any lumps?**	1. Yes<br>2. No<br>9. Doesn't know	 → Next symptom<br>→ Next symptom	
**7108**	**Did (s)he have any lumps on the neck?**	1. Yes<br>2. No<br>9. Doesn't know		
**7109**	**Did (s)he have any lumps on the armpit?**	1. Yes<br>2. No<br>9. Doesn't know		
**7110**	**Did (s)he have any lumps on the groin?**	1. Yes<br>2. No<br>9. Doesn't know		

**7111**	**Was (s)he in any way paralysed?**	1. Yes<br>2. No<br>9. Doesn't know	 → 7114<br>→ 7114	
**7112**	**Did (s)he have paralysis of only one side of the body?**	1. Yes<br>2. No<br>9. Doesn't know		
**7113**	**Which were the limbs or body parts paralysed?**<br>*Enter more than one if applicable.*	1. Right side<br>2. Left side<br>3. Lower part of body<br>4. Upper part of body<br>5. One leg only<br>6. One arm only<br>7. Whole body<br>8. Other		Multi-select
**7118**	**Did (s)he have yellow discoloration of the eyes?**	1. Yes<br>2. No<br>9. Doesn't know	 → Next symptom<br>→ Next symptom	
**7119**	**For how long did (s)he have the yellow discoloration?**<br>*Less than 1 day = '0'.*	1. Days __ __<br>2. Months __ __<br>- Doesn't know		Days,Months=<Age at Death<br>Select<br>1- [0:30]<br>2- [1:60]
**7124**	**Was the baby able to suckle or bottle-feed within the first 24 hours after birth?**<br>*Ask only if the child was <1 year old at death.*	1. Yes<br>2. No<br>9. Doesn't know		Enabled only for child <1 year
**7125**	**Did the baby ever suckle in a normal way?**<br>*Ask only if the child was <1 year old at death.*	1. Yes<br>2. No<br>9. Doesn't know		Enabled only for child <1 year
**7126**	**Did the baby stop suckling?**<br>*Ask only if the child was <1 year old at death.*	1. Yes<br>2. No<br>9. Doesn't know	 → 7129<br>→ 7129	Enabled only for child <1 year
**7128**	**How long after birth did the baby stop suckling?**<br>*Less than 1 day = '0'.*<br>*Ask only if the child was <1 year old at death.*	1. Days __ __<br>2. Months __ __<br>- Doesn't know		Select<br>1- [0:30]<br>2- [1:11]<br>Enabled only for child <1 year
**7129**	**Did the baby have convulsions starting within the first 24 hours of life?**<br>*Ask only if the child was <1 year old at death.*	1. Yes<br>2. No<br>9. Doesn't know	→ 7131	Enabled only for child <1 year

**7130**	**Did the baby have convulsions starting more than 24 hours after birth?**<br>*Ask only if the child was <1 year old at death.*	1. Yes<br>2. No<br>9. Doesn't know		Enabled only for child <1 year
**7131**	**Did the baby's body become stiff, with the back arched backwards?**	1. Yes<br>2. No<br>9. Doesn't know		Enabled only for child <1 year
**7132**	**During the illness that led to death, did the baby have a bulging or raised fontanelle?**<br>*Ask only if the child was ≤18 months old at death. Show photo (if available).*	1. Yes<br>2. No<br>9. Doesn't know	→ 7134	Enabled only for child ≤18 months
**7133**	**During the illness that led to death, did the baby have a sunken fontanelle?**<br>*Ask only if the child was ≤18 months old at death. Show photo (if available).*	1. Yes<br>2. No<br>9. Doesn't know		Enabled only for child ≤18 months
**7134**	**During the illness that led to death, did the baby become unresponsive or unconscious?**<br>*Ask only if the child was <1 year old at death.*	1. Yes<br>2. No<br>9. Doesn't know	 → 7217<br>→ 7217	Enabled only for child <1 year
**7135**	**Did the child become unresponsive or unconscious soon after birth, within less than 24 hours?**<br>*Ask only if the child was <1 year old at death.*	1. Yes<br>2. No<br>9. Doesn't know	→ 7217	Enabled only for child <1 year
**7136**	**Did the child become unresponsive or unconscious more than 24 hours after birth?**<br>*Ask only if the child was <1 year old at death.*	1. Yes<br>2. No<br>9. Doesn't know		Enabled only for child <1 year
**7217**	**At birth, was the baby of usual size?**<br>*Ask only if the child was <1 year old at death. Show photos (if available).*	1. Yes<br>2. No<br>9. Doesn't know	→ 7221	Enabled only for child <1 year
**7218**	**At birth, was the baby smaller than usual (weighing under 2.5 kg)?**	1. Yes<br>2. No<br>9. Doesn't know	 → 7220<br>→ 7220	
**7219**	**At birth, was the baby very much smaller than usual (weighing under 1 kg)?**	1. Yes<br>2. No<br>9. Doesn't know	→ All: 7221	
**7220**	**At birth, was the baby larger than usual (weighing over 4.5 kg)?**	1. Yes<br>2. No<br>9. Doesn't know		

**7221**	**What was the weight (in grams) of the deceased at birth?**<br>*Respondents may give the answer in kilograms. For the data entry, convert to grams. 1 kilogram=1,000 grams.*<br>*Ask only if the child was <1 year old at death.*	Grams __ __ __ __<br>- Doesn't know		[1000:5000]<br>Enabled only for child <1 year
**7225**	**Was any part of the child physically abnormal at time of delivery? (for example: body part too large or too small, additional growth on the body)**<br>*Ask only if the child was <1 year old at death.*	1. Yes<br>2. No<br>9. Doesn't know	 → 7210	Enabled only for child <1 year
**7226**	**Did the child have a swelling or defect on the back at time of birth?**<br>*Ask only if the child was <1 year old at death.*	1. Yes<br>2. No<br>9. Doesn't know		
**7227**	**Did the baby/child have a very large head at time of birth?**<br>*Ask only if the child was <1 year old at death.*	1. Yes<br>2. No<br>9. Doesn't know	→ 7210	
**7228**	**Did the baby/child have a very small head at time of birth?**<br>*Ask only if the child was <1 year old at death.*	1. Yes<br>2. No<br>9. Doesn't know		
**7210**	**Was the child part of a multiple birth?**<br>*Ask only if the child was <1 year old at death. If two or more children are born at the same time, it is counted as a multiple birth, even if one or more of the babies are born dead.*	1. Yes<br>2. No<br>9. Doesn't know	 → 7212<br>→ 7212	Enabled only for child <1 year
**7211**	**Was the child the first, second, or later in the birth order?**<br>*Ask only if the child was <1 year old at death.*	1. First<br>2. Second or later<br>9. Doesn't know		Select- only one<br>Enabled only for child <1 year
**7212**	**Is the mother still alive?**<br>*If the mother is present at the interview, select 'yes' without asking the question aloud. Only read this question if the respondent is not the mother and if it is not yet known if the mother is alive.*<br>*Ask only if the child was <1 year old at death.*	1. Yes<br>2. No<br>9. Doesn't know	→ 7215<br><br>→ 7215	Enabled only for child <1 year
**7213**	**Did the mother die before, during or after the delivery?**<br>*Ask only if the child was <1 year old at death.*	1. Before delivery<br>2. During delivery<br>3. After delivery<br>9. Doesn't know	→ 7215<br>→ 7215<br><br>→ 7215	Enabled only for child <1 year

**7214**	**How long after the delivery did the mother die?**<br>*Ask only if the child was <1 year old at death. If less than 24 hours, record '0' days. If <1 week, record in days; if <2 months, record in weeks; if ≥ 2 months, record in completed months.*	1. Days __<br>2. Weeks __<br>3. Months __ __<br>- Doesn't know		Select<br>1- [0:6]<br>2- [1:7]<br>3- [2:60]<br>Enabled only for child <1 year
**7215**	**Where was the deceased born?**<br>*Ask only if the child was <1 year old at death. Read the question and slowly read the first 5 choices. Respondent should hear all 5 choices and then respond.*	1. Hospital<br>2. Other health facility<br>3. Home of the deceased<br>4. On route to hospital or facility<br>5. Other<br>9. Doesn't know		Select- only one<br>Enabled only for child <1 year
**7216**	**Did you/the mother receive professional assistance during the delivery?**<br>*Ask only if the child was <1 year old at death. Explain to the respondent what is meant by professional assistance: delivery attended by a medical professional (doctor, nurse or midwife).*	1. Yes<br>2. No<br>9. Doesn't know		Enabled only for child <1 year<br><br>If respondent's sex (Q1016) is "Female" and relationship to the deceased (Q2006) is "Parent", use "you"; otherwise, use "the mother"
**7222**	**How many months long was the pregnancy before the child was born?**<br>*Ask only if the child was <1 year old at death.*	Months __ __<br>- Doesn't know		[6:10]<br>Enabled only for child <1 year
**7223**	**Were there any complications in the late part of the pregnancy (defined as the last 3 months, before labour)?**<br>*Ask only if the child was <1 year old at death.*	1. Yes<br>2. No<br>9. Doesn't know		Enabled only for child <1 year
**7224**	**Were there any complications during labour or delivery?**<br>*Ask only if the child was <1 year old at death.*	1. Yes<br>2. No<br>9. Doesn't know		Enabled only for child <1 year
**7229**	**How many births, including stillbirths, did the baby's mother have before this baby?**	Births __ __<br>- Doesn't know		[0:20]

<table>
<tr><td colspan="5">KEY SYMPTOMS</td></tr>
<tr><td>11001</td><td>You said that (s)he had the following symptoms.
[List of positive symptoms]
Which one occurred first? Second? [Continue until all symptoms have been put in order.]
If ‘1’ was previously selected for only one symptom, do not ask this question. If respondent does not know the order, enter the symptoms in the order they were asked about.</td><td>1.
2.
3.
4.
5.
6.</td><td></td><td>Enabled only if ‘1. Yes’ was selected for more than one symptom
All important positive symptoms appear, and the program requests the surveyor to put them in chronological order</td></tr>
<tr><td></td><td colspan="3">Thank you for the information. You mentioned that the deceased had some problems before death. Can you please tell me about any medical treatment that they received for these problems?
USE THIS SPACE to take notes on details of medical treatment that the deceased received during the illness that led to death, as well as any additional probing of positive symptoms during the narrative. For paper forms, refer to the reminders (attached separately) for additional information to probe for each positive symptom.
____
____
____
____________________</td><td>On left-hand side, summary of answers from probing of all positive symptoms appears in chronological order.

Recording begins at the start of this section.</td></tr>
<tr><td>11002</td><td colspan="3">[Narrative] Thank you for your information. Now can you please tell me in your own words about the events that led to the death?
Using the notes, read out the organised history to the respondent and get his/her confirmation before writing it below.
Excuse me for a few minutes while I write out what you have told me. During this time, you are free to do something else for a few minutes. Afterwards, I have just a few more questions for you and we will close the interview. I appreciate your patience.
Please put in chronological order the key symptoms, any treatments received, and probing questions. Remember your goal is NOT to arrive at a diagnosis (that will be done by the central doctors) but the KEY symptoms and the KEY sickness history in chronological order.
____
____
____
____
____
____
____
____
____
____________________</td><td>Minimum of 300 characters to be entered in the narrative text box

Recording ends after completion of this question.</td></tr>
</table>

**I would like to ask some questions about the treatments that the deceased received and health services that were accessed.**				
**9001**	**Did (s)he receive any treatment for the illness that led to death?**	1. Yes<br>2. No<br>9. Doesn't know	 → 9011<br>→ 9011	
**9002**	**Did (s)he receive oral rehydration salts?**	1. Yes<br>2. No<br>9. Doesn't know		
**9003**	**Did (s)he receive (or need) intravenous fluids (drip) treatment?**	1. Yes<br>2. No<br>9. Doesn't know		
**9006**	**Did (s)he receive (or need) injectable antibiotics?**	1. Yes<br>2. No<br>9. Doesn't know		
**9008**	**Did (s)he receive (or need) an operation for the illness?**	1. Yes<br>2. No<br>9. Doesn't know	 → 9011<br>→ 9011	
**9009**	**Did (s)he have the operation within 1 month before death?**	1. Yes<br>2. No<br>9. Doesn't know		
**9011**	**Did (s)he receive any immunizations?**	1. Yes<br>2. No<br>9. Doesn't know	 → 9015<br>→ 9015	
**9012**	**Do you have the child's vaccination card?**	1. Yes<br>2. No	 → 9015	
**9013**	**Can I see the vaccination card?**	1. Yes<br>2. No	 → 9015	
**9014**	[Select EPI vaccines done.]	1. BCG<br>2. DPT 1,2,3<br>3. Hep B<br>4. Hib<br>5. Meningitis<br>6. Penta 1,2,3<br>7. Pneumo<br>8. Polio 1,2,3<br>10. Rota<br>11. No vaccines<br>9. Doesn't know		multiselect

**9015**	**Was care sought outside the home while (s)he had this illness?**	1. Yes<br>2. No<br>9. Doesn't know	 → 9018<br>→ 9018	
**9016**	**Where or from whom did you seek this care?**<br>*Enter more than one if applicable.*	1. Government Hospital<br>2. Government Health centre or clinic<br>3. Private Hospital<br>4. Ayush (Ayurveda, Unani, etc.)<br>5. Relative, friend (outside household)<br>6. Other<br>9. Doesn't know		multiselect
**9018**	**Did a health care worker tell you the cause of death?**	1. Yes<br>2. No<br>9. Doesn't know	 → 9034<br>→ 9034	
**9019**	**What did the health care worker say?**			text
**9034**	**Over the course of illness, did the total costs of care and treatment interfere with other routine household expenses?**	1. Yes<br>2. No<br>9. Doesn't know		
**9035**	**Did the household have to borrow money or sell any household items to pay for treatment during the illness?**	1. Yes<br>2. No<br>9. Doesn't know		
**9036**	**Was the death registered in the local government system (municipal or civil registration)?**	1. Yes<br>2. No<br>9. Doesn't know		
**9037**	**Did you receive a doctor's death certificate with the cause of death?**	1. Yes<br>2. No<br>9. Doesn't know	 → 12098<br>→ 12098	
**9038**	**Can I see the death certificate?**	1. Yes<br>2. No	 → 12098	
**9038a**	[Enter the cause of death.]			text
**9038b**	[Enter the date on the certificate.]	1. Day __ __<br>2. Month __ __<br>3. Year __ __ __ __		1- [1:31]<br>2- [1:12]<br>3- [2019:2022]
**Before I conclude, two short questions for you:**				
**12098**	**Would you say the interview was:**	1. Too long<br>2. Too short<br>3. About right length<br>9. No comment		Select- only one
**12099**	**Do you have any suggestions on how we can do better?**			text,maxlength[200]
NOTE: THE FOLLOWING QUESTIONS ARE FOR AUTO-POPULATION TO COMPLETE AND ARE NOT TO BE ASKED TO RESPONDENTS.				

**13004**	[Name of VA interviewer]			text
**13005**	[Time at start of interview]	[hh: __ __mm __ __]		
**13006**	[Date of interview]	[dd/mm/yyyy]		
**13021**	[Time at end of interview]	[hh: __ __mm __ __]		
