# Supplementary material for: The mortality burden from COVID in low-income settings: evidence from verbal autopsies in India
Source: BMC Public Health. 2026 Jan 22;26:1567. doi: 10.1186/s12889-026-26215-9 (PMC13188735; doi:10.1186/s12889-026-26215-9)
Supplement: Supplementary file 4 — Supplementary Material 4. [file 12889_2026_26215_MOESM4_ESM.pdf]

CGHR Study Neonate e-VA Instrument
Questionnaire for the death of a child aged under 28 days

<u>Instructions for use of the tool</u>
Questions to be read to the respondent are in bold.
[Questions that are NOT to be read to respondent are in brackets.]
Hints to the interviewer are in italic text below relevant questions. These are only hints for the interviewer and are NOT to be read to respondents.

ID	Question	Answer(s)	Skip To	Programming Notes
Before beginning the survey, ensure that the respondent is an adult.<br>**Hello, my name is** [surveyor's name] **and I am working for CMIE. We are studying ways to improve the health status of the community. We very much appreciate your participation in this survey. As part of the survey, we invite you to provide information on any deaths that happened in your household. We will ask questions related to the illness of the deceased and some other questions. This will help us understand the patterns of deaths, and will help establish how to improve health in your city/village.**				
**1005**	**What is your** [the respondent's] **MEM_ID?**	1. [Household MEM_ID list]<br>2. Someone else	→ 1006	Select- only one
**1005a**	[Please enter an identifier for the person (name, ID, etc.)]			text
**1006**	[Choose the respondent's sex.]	1. Male<br>2. Female<br>3. Transgender		Select- only one
**1007**	**How old are you in completed years?**<br>*Record the respondent's age as of her/his last birthday. Record '99' if don't know the age.*	Years __ __		[18:99]
**1016**	**What is the highest grade you have completed?**<br>*<1 year: '0', grade if only up to school level: 1-12, college/university attended but not graduated: '13', graduated: '14', never attended: '98', not known: '99'*	Grade __ __		[0:14,98:99]
**13015**	**What is your marital status?**	1. Married<br>2. Married, but Gauna not performed<br>3. Widowed<br>4. Divorced<br>5. Separated<br>6. Deserted<br>7. Never married<br>8. Engaged<br>9. Doesn't know		Select- only one
**I would like to tell you more about the study and obtain your consent.** [Read the consent form to the family.]				
**1017**	[Did the respondent give consent?]	1. Yes<br>2. No	→ 2003	

**1017a**	[Why did the respondent, in their own words, not want to do the interview?]		→ End the Interview	text,maxlength[100]
**I would now like to ask you some detailed questions about any neonate deaths that have occurred in the house since January 1, 2019.**				Display name, sex, and age (with units) of deceased from enumeration database before the following questions
**2003**	**What was the sex of the deceased?**	1. Male<br>2. Female		Select- only one
**2004**	**When was the deceased born?**	1. Day __ __<br>2. Month __ __<br>3. Year __ __ __ __		1- [1:31]<br>2- [1:12]<br>3- [2019-age-5%:2021-age+5%]
**2005**	**When did (s)he die?**	1. Day __ __<br>2. Month __ __<br>3. Year __ __ __ __		1- [1:31]<br>2- [1:12]<br>3- [2019:2021]<br>Death date > Birth date
**2006**	**What is your/the respondent's relationship to the deceased?**	1. Parent<br>2. Brother/sister<br>3. Other family member<br>4. Friend<br>5. Another relationship<br>9. None of the above		Select- only one
**2007**	**Did you/the respondent live with the deceased in the period leading to her/his death?**	1. Yes<br>2. No		
2008	**Where did the deceased die?**	1. Hospital<br>2. Other health facility<br>3. Home<br>4. On route to hospital or facility<br>5. Other<br>9. Doesn't know		Select- only one
**3001**	**What do you (the respondent) think the deceased died of?**<br>*Record the respondent's own exact words.*			text

**3002**	[Select what you believe the respondent reported as the cause of death. If they do not know, then enter "Does not know".]	1. Prematurity / Low birth weight<br>2. Infections<br>3. Birth asphyxia / Birth trauma<br>4. Congenital problems<br>5. Diarrhoea<br>6. Pneumonia<br>7. Injury/accident<br>8. Other or unknown cause		Based on the respondent's stated COD captured here, apply symptom sequence (attached separately) to determine the order of the questions in Sections 5 & 7<br><br>In the eVA, options will be suggested as the surveyor types, which are then automatically classified into one of these categories.
**4001**	**Did the baby ever cry?**	1. Yes<br>2. No<br>9. Doesn't know	 → 4006<br>→ 4006	
**4002**	**Did the baby cry immediately after birth, even if only a little bit?**	1. Yes<br>2. No<br>9. Doesn't know		
**4003**	**How many minutes after birth did the baby first cry?**	Minutes __ __<br>- Doesn't know		Minutes=<Age at Death [1:60]
**4004**	**Did the baby stop being able to cry?**	1. Yes<br>2. No<br>9. Doesn't know	 → 4006<br>→ 4006	
**4005**	**How many hours before death did the baby stop crying?**	Hours __ __<br>- Doesn't know		Hours=<Age at Death [1:24]
**4006**	**Did the baby ever move?**	1. Yes<br>2. No<br>9. Doesn't know		
**4007**	**Did the baby ever breathe?**	1. Yes<br>2. No<br>9. Doesn't know	 → 4012<br>→ 4012	If the respondent answered "No" or "Doesn't know" to both of Q4001 and Q4006, the skips change to 4011
**4008**	**Did the baby breathe immediately after birth, even a little?**	1. Yes<br>2. No<br>9. Doesn't know		
**4009**	**Did the baby have a breathing problem?**	1. Yes<br>2. No<br>9. Doesn't know		

**4010**	**Was the baby given assistance to breathe at birth?**	1. Yes<br>2. No<br>9. Doesn’t know	→ All: 4012	
**4011**	**If the baby didn't show any sign of life, was it born dead?**	1. Yes<br>2. No<br>9. Doesn’t know		
**4012**	**Were there any bruises or signs of injury on the baby's body after the birth?**	1. Yes<br>2. No<br>9. Doesn’t know	→ All: 5001	If the respondent answered “Yes” to Q4011, no skip
**4013**	**Was the baby's body soft, pulpy and discoloured and the skin peeling away?**	1. Yes<br>2. No<br>9. Doesn’t know	→ All: 7210	
**5001**	**Did (s)he suffer from any injury or accident that led to her/his death?**	1. Yes<br>2. No<br>9. Doesn’t know	 → 6001<br>→ 6001	
**5002**	**What was the nature of the injury or accident?**	1. Road traffic accident<br>2. Non-road transport accident<br>3. Drowning<br>4. Fall<br>5. Injury by falling object / blunt force<br>6. Pesticide poisoning<br>7. Other poisoning<br>8. Electrocution<br>10. Burns/fire<br>11. Injured by a firearm<br>12. Bite or sting<br>13. Stabbed/cut/pierced<br>14. Strangled<br>15. Flood<br>16. Earthquake<br>17. Other<br>99. Doesn’t know	→ 5007<br><br><br><br><br><br><br><br><br><br>→ 5009<br><br><br>→ Next section<br>→ Next section	multiselect
**5003**	**Was (s)he subject to violence (homicide, abuse)?**	1. Yes<br>2. No<br>9. Doesn’t know		
**5004**	**Was the injury accidental?**	1. Yes<br>2. No<br>9. Doesn’t know	→ Next section	

**5006**	**Was the injury intentionally inflicted by someone else?**	1. Yes<br>2. No<br>9. Doesn’t know	→ All: Next section	
**5007**	**What was her/his role in the road traffic accident?**	1. Pedestrian<br>2. Passenger in car or light vehicle<br>3. Passenger in bus or heavy vehicle<br>4. Passenger on a motorcycle<br>5. Passenger on a pedal cycle<br>6. Other<br>9. Doesn’t know		Select- only one
**5008**	**What was the counterpart that was hit during the road traffic accident?**	1. Pedestrian<br>2. Stationary object<br>3. Car or light vehicle<br>4. Bus or heavy vehicle<br>5. Motorcycle<br>6. Pedal cycle<br>7. Other<br>9. Doesn’t know	→ All: Next section	Select- only one
**5009**	**What was the animal/insect?**	1. Dog<br>2. Snake<br>3. Insect or scorpion<br>4. Other<br>9. Doesn’t know		Select- only one
**6002**	**Before the illness that led to death, was the baby growing normally?**	1. Yes<br>2. No<br>9. Doesn’t know		
**6003**	**For how many days was (s)he ill before death?**<br>*Less than 1 day = ‘0’.*	Days __ __<br>- Doesn’t know		Days=<Age at Death [0:27]
**6005**	**Did (s)he die suddenly?**<br>*Suddenly means died unexpectedly within 24 hours of being in regular health*	1. Yes<br>2. No<br>9. Doesn’t know		
**7001**	**Did (s)he have a fever?**	1. Yes<br>2. No<br>9. Doesn’t know	 → Next section<br>→ Next section	
**7002**	**How many days did the fever last?**<br>*Less than 1 day = ‘0’.*	Days __ __<br>- Doesn’t know		Days=<Age at Death [0:27]

**7004**	**Did the fever continue until death?**	1. Yes<br>2. No<br>9. Doesn't know		
**7008**	**Did (s)he have a cough?**	1. Yes<br>2. No<br>9. Doesn't know	 → Next section<br>→ Next section	
**7013**	**Did (s)he make a whooping sound when coughing?**	1. Yes<br>2. No<br>9. Doesn't know		
**7015**	**Did (s)he have any difficulty breathing?**	1. Yes<br>2. No<br>9. Doesn't know	 → 7019<br>→ 7019	
**7016**	**For how many days did the difficulty breathing last?**<br>*Less than 1 day = '0'.*	Days __ __<br>- Doesn't know		Days=<Age at Death<br>[0:27]
**7019**	**During the illness that led to death, did (s)he have fast breathing?**	1. Yes<br>2. No<br>9. Doesn't know	 → 7022<br>→ 7022	
**7020**	**For how many days did the fast breathing last?**<br>*Less than 1 day = '0'.*	Days __ __<br>- Doesn't know		Days=<Age at Death<br>[0:27]
**7022**	**Did (s)he have breathlessness?**	1. Yes<br>2. No<br>9. Doesn't know	 → 7027<br>→ 7027	
**7023**	**For how many days did (s)he have breathlessness?**<br>*Less than 1 day = '0'.*	Days __ __<br>- Doesn't know		Days=<Age at Death<br>[0:27]
**7027**	**Did you see the lower chest wall/ribs being pulled in as the child breathed in?**<br>*Show photos/video (if available).*	1. Yes<br>2. No<br>9. Doesn't know		
**7028**	**During the illness that led to death did his/her breathing sound like any of the following:**	1. Stridor<br>2. Grunting<br>3. Wheezing<br>4. None of the above<br>9. Doesn't know		Select- only one
**7034**	**Did (s)he have more frequent loose or liquid stools than usual?**<br>*Ask the respondent about his/her understanding of what diarrhoea is (having more frequent loose or liquid stools than usual); if unclear or wrong, explain to the respondent what diarrhoea is.*	1. Yes<br>2. No<br>9. Doesn't know	 → 7040<br>→ 7040	

**7036**	**How many stools did the baby have on the day that loose or liquid stools were most frequent?**	No. of stools: __ __<br>- Doesn’t know		[1:25]
**7037**	**How many days before death did the frequent loose or liquid stools start?**<br>*Less than 1 day = ‘0’.*	Days __ __<br>- Doesn’t know		Days=<Age at Death<br>[0:27]
**7039**	**Did the frequent loose or liquid stools continue until death?**	1. Yes<br>2. No<br>9. Doesn’t know		
**7040**	**At any time during the final illness was there blood in the stools?**	1. Yes<br>2. No<br>9. Doesn’t know		
**7042**	**Did (s)he vomit?**	1. Yes<br>2. No<br>9. Doesn’t know	 → Next section<br>→ Next section	
**7043**	**To clarify: Did (s)he vomit in the week preceding death?**	1. Yes<br>2. No<br>9. Doesn’t know		
**7065**	**Was (s)he unconscious during the illness that led to death?**	1. Yes<br>2. No<br>9. Doesn’t know	 → Next section<br>→ Next section	
**7066**	**Was (s)he unconscious for more than 24 hours before death?**	1. Yes<br>2. No<br>9. Doesn’t know		
**7070**	**Did (s)he have convulsions?**	1. Yes<br>2. No<br>9. Doesn’t know		
**7085**	**During the illness that led to death, did (s)he have any skin rash?**	1. Yes<br>2. No<br>9. Doesn’t know		
**7091**	**During the illness that led to death did (s)he have areas of skin that turned black?**	1. Yes<br>2. No<br>9. Doesn’t know		
**7092**	**During the illness that led to death did (s)he have areas of the skin with redness and swelling?**	1. Yes<br>2. No<br>9. Doesn’t know		
**7093**	**During the illness that led to death did (s)he bleed from anywhere?**	1. Yes<br>2. No<br>9. Doesn’t know		

**7118**	**Did (s)he have yellow discoloration of the eyes?**	1. Yes<br>2. No<br>9. Doesn't know		
**7124**	**Was the baby able to suckle or bottle-feed within the first 24 hours after birth?**	1. Yes<br>2. No<br>9. Doesn't know		
**7125**	**Did the baby ever suckle in a normal way?**	1. Yes<br>2. No<br>9. Doesn't know		
**7126**	**Did the baby stop suckling?**	1. Yes<br>2. No<br>9. Doesn't know	 → 7129<br>→ 7129	
**7127**	**How many days after birth did the baby stop suckling?**<br>*Less than 1 day = '0'.*	Days __ __<br>- Doesn't know		Days=<Age at Death [0:27]
**7129**	**Did the baby have convulsions starting within the first 24 hours of life?**	1. Yes<br>2. No<br>9. Doesn't know	→ 7131	
**7130**	**Did the baby have convulsions starting more than 24 hours after birth?**	1. Yes<br>2. No<br>9. Doesn't know		
**7131**	**Did the baby's body become stiff, with the back arched backwards?**	1. Yes<br>2. No<br>9. Doesn't know		
**7132**	**During the illness that led to death did the baby have a bulging or raised fontanelle?**<br>*Show photo (if available).*	1. Yes<br>2. No<br>9. Doesn't know	→ 7134	
**7133**	**During the illness that led to death did the baby have a sunken fontanelle?**<br>*Show photo (if available).*	1. Yes<br>2. No<br>9. Doesn't know		
**7134**	**During the illness that led to death, did the baby become unresponsive or unconscious?**	1. Yes<br>2. No<br>9. Doesn't know	 → 7137<br>→ 7137	
**7135**	**Did the baby become unresponsive or unconscious soon after birth, within less than 24 hours?**	1. Yes<br>2. No<br>9. Doesn't know	→ 7137	

**7136**	**Did the baby become unresponsive or unconscious more than 24 hours after birth?**	1. Yes<br>2. No<br>9. Doesn't know		
**7137**	**During the illness that led to death, did the baby become cold to touch?**	1. Yes<br>2. No<br>9. Doesn't know	 → 7139<br>→ 7139	
**7138**	**How many days old was the baby when it started feeling cold to touch?**	Days __ __<br>- Doesn't know		Days=<Age at Death [0:27]
**7139**	**During the illness that led to death, did the baby become lethargic after a period of normal activity?**	1. Yes<br>2. No<br>9. Doesn't know		
**7140**	**Did the baby have redness or pus drainage from the umbilical cord stump?**	1. Yes<br>2. No<br>9. Doesn't know		
**7141**	**During the illness that led to death, did the baby have skin ulcer(s) or pits?**	1. Yes<br>2. No<br>9. Doesn't know		
**7142**	**During the illness that led to death, did the baby have yellow skin, palms (hand) or soles (foot)?**	1. Yes<br>2. No<br>9. Doesn't know		
**7143**	**Did the baby or infant appear to be healthy and then just die suddenly?**<br>*Suddenly means died unexpectedly within 24 hours of being in regular health*	1. Yes<br>2. No<br>9. Doesn't know		
**7217**	**At birth, was the baby of usual size?**<br>*Show photos (if available).*	1. Yes<br>2. No<br>9. Doesn't know	→ 7221	
**7218**	**At birth, was the baby smaller than usual (weighing under 2.5 kg)?**	1. Yes<br>2. No<br>9. Doesn't know	 → 7220<br>→ 7220	
**7219**	**At birth, was the baby very much smaller than usual (weighing under 1 kg)?**	1. Yes<br>2. No<br>9. Doesn't know	→ All: 7221	
**7220**	**At birth, was the baby larger than usual (weighing over 4.5 kg)?**	1. Yes<br>2. No<br>9. Doesn't know		
**7221**	**What was the weight (in grams) of the deceased at birth?**<br>*Respondents may give the answer in kilograms. For the data entry, convert to grams. 1 kilogram=1,000 grams.*	Grams __ __ __ __<br>- Doesn't know		[1000:5000]

**7225**	**Was any part of the baby physically abnormal at time of delivery? (for example: body part too large or too small, additional growth on the body)**	1. Yes<br>2. No<br>9. Doesn't know	 → Next section 	
**7226**	**Did the child have a swelling or defect on the back at time of birth?**	1. Yes<br>2. No<br>9. Doesn't know		
**7227**	**Did the baby/child have a very large head at time of birth?**	1. Yes<br>2. No<br>9. Doesn't know	→ Next section	
**7228**	**Did the baby/child have a very small head at time of birth?**	1. Yes<br>2. No<br>9. Doesn't know		
**7210**	**Was the child part of a multiple birth?**<br>*If two or more babies are born at the same time, it is counted as a multiple birth, even if one or more of the babies are born dead.*	1. Yes<br>2. No<br>9. Doesn't know	 → 7212<br>→ 7212	
**7211**	**Was the baby the first, second, or later in the birth order?**	1. First<br>2. Second or later<br>9. Doesn't know		Select- only one
**7212**	**Is the mother still alive?**<br>*If the mother is present at the interview, select 'yes' without asking the question aloud. Only read this question if the respondent is not the mother and if it is not yet known if the mother is alive.*	1. Yes<br>2. No<br>9. Doesn't know	→ 7215<br><br>→ 7215	
**7213**	**Did the mother die before, during or after the delivery?**	1. Before delivery<br>2. During delivery<br>3. After delivery<br>9. Doesn't know	→ 7215<br>→ 7215<br><br>→ 7215	
**7214**	**How long after the delivery did the mother die?**<br>*If less than 24 hours, record '0' days. If <1 week, record in days; if <2 months, record in weeks; if ≥ 2 months, record in completed months.*	1. Days __ __<br>2. Weeks __<br>3. Months __ __<br>- Doesn't know		Select<br>1- [0:6]<br>2- [1:7]<br>3- [2:60]
**7215**	**Where was the deceased born?**<br>*Read the question and slowly read the first 5 choices. Respondent should hear all 5 choices and then respond.*	1. Hospital<br>2. Other health facility<br>3. Home<br>4. On route to hospital or facility<br>5. Other<br>9. Doesn't know		Select- only one

**7216**	**Did you/the mother receive professional assistance during the delivery?**<br>*Explain to the respondent what is meant by professional assistance: delivery attended by a medical professional (doctor, nurse or midwife).*	1. Yes<br>2. No<br>9. Doesn't know		For all questions with "you/the [baby's] mother", if respondent's sex (Q1016) is "Female" and relationship to the deceased (Q2006) is "Parent", use "you"; otherwise, use "the mother"
**7222**	**How many months long was the pregnancy before the child was born?**	Months __ __<br>- Doesn't know		[6:10]
**7223**	**Were there any complications in the late part of the pregnancy (defined as the last 3 months before labour)?**	1. Yes<br>2. No<br>9. Doesn't know		
**7224**	**Were there any complications during labour or delivery?**	1. Yes<br>2. No<br>9. Doesn't know		
**7229**	**How many births, including stillbirths, did the baby's mother have before this baby?**	Births __ __<br>- Doesn't know		[0:20]
**7230**	**Was the baby moving in the last few days before the birth?**	1. Yes<br>2. No<br>9. Doesn't know		
**7231**	**Did the baby stop moving in the womb before labour started?**	1. Yes<br>2. No<br>9. Doesn't know	 → 7233<br>→ 7233	
**7232**	**How long before labour did you / the mother** (if respondent is another person who examined the mother) **last feel the baby move?**<br>*Enter duration in hours or days. If less than one hour, enter '0'.*	1. Hours __ __<br>2. Days __ __<br>- Doesn't know		Select<br>1- [0:24]<br>2- [1:30]
**7233**	**How many hours did labour and delivery take?**<br>*If less than one hour enter '0'.*	Hours __ __<br>- Doesn't know		[0:72]
**7234**	**Was the baby born 24 hours or more after the water broke?**	1. Yes<br>2. No<br>9. Doesn't know		
**7235**	**Was the liquid foul smelling?**	1. Yes<br>2. No<br>9. Doesn't know		
**7236**	**What was the colour of the liquid when the water broke?**	1. Green or brown<br>2. Clear (normal)<br>3. Other<br>9. Doesn't know		Select- only one

**7237**	**Was the delivery normal vaginal, without forceps or vacuum?**	1. Yes<br>2. No<br>9. Doesn't know	→ 7239	
**7238**	**Was the delivery vaginal, with forceps or vacuum?**	1. Yes<br>2. No<br>9. Doesn't know		
**7239**	**Did you/the mother receive any vaccinations since reaching adulthood including during this pregnancy?**	1. Yes<br>2. No<br>9. Doesn't know	 → 7242<br>→ 7242	
**7240**	**How many doses?**	Doses __ __<br>- Doesn't know		[0:7]
**7241**	**Did you/the mother receive tetanus toxoid (TT) vaccine?**	1. Yes<br>2. No<br>9. Doesn't know		
**7242**	**During labour, did the baby's mother suffer from fever?**	1. Yes<br>2. No<br>9. Doesn't know		
**7243**	**During the last 3 months of pregnancy, labour or delivery, did you/the baby's mother suffer from high blood pressure?**	1. Yes<br>2. No<br>9. Doesn't know		
**7244**	**Did you/the baby's mother have diabetes mellitus?**	1. Yes<br>2. No<br>9. Doesn't know		
**7245**	**Did you/the baby's mother have foul smelling vaginal discharge during pregnancy or after delivery?**	1. Yes<br>2. No<br>9. Doesn't know		
**7246**	**During the last 3 months of pregnancy, labour or delivery, did you/the baby's mother suffer from convulsions?**	1. Yes<br>2. No<br>9. Doesn't know		
**7247**	**During the last 3 months of pregnancy did you/the baby's mother suffer from blurred vision?**	1. Yes<br>2. No<br>9. Doesn't know		
**7248**	**Did you/the baby's mother have severe anemia?**	1. Yes<br>2. No<br>9. Doesn't know		
**7249**	**Did you/the baby's mother have vaginal bleeding during the last 3 months of pregnancy but before labour started?**	1. Yes<br>2. No<br>9. Doesn't know		

**7250**	**Did the baby's bottom, feet, arm or hand come out of the vagina before its head?**	1. Yes<br>2. No<br>9. Doesn't know		
**7251**	**Was the umbilical cord wrapped more than once around the neck of the child at birth?**	1. Yes<br>2. No<br>9. Doesn't know		
**7252**	**Was the umbilical cord delivered first?**	1. Yes<br>2. No<br>9. Doesn't know		
**7253**	**Was the baby blue in colour at birth?**	1. Yes<br>2. No<br>9. Doesn't know		
**KEY SYMPTOMS**				
**11001**	**You said that (s)he had** [list positive symptoms]**. Which one occurred first? Second?** [Continue until all symptoms have been put in order.]<br>*If '1' was previously selected for only one symptom, do not ask this question. If respondent does not know the order, enter the symptoms in the order they were asked about.*	1.<br>2.<br>3.<br>4.<br>5.<br>6.		Enabled only if '1. Yes' was selected for more than one symptom<br>All positive symptoms appear, and the program requests the surveyor to put them in chronological order
	**Thank you for the information. You mentioned that the deceased had some problems before death. Can you please tell me about any medical treatment that they received for these problems?**<br>*USE THIS SPACE to take notes on details of medical treatment that the deceased received during the illness that led to death, as well as any additional probing of positive symptoms during the narrative. For paper forms, refer to the reminders (attached separately) for additional information to probe for each positive symptom.*<br>____<br>____<br>____<br>____<br>____<br>____<br>____<br>____<br>____<br>____<br>____			On left-hand side, summary of answers from probing of all positive symptoms appears in chronological order.<br><br>Recording begins at the start of this section.

**11002**	[Narrative] **Thank you for your information. Now can you please tell me in your own words about the events that led to the death?**<br>*Using the notes, read out the organised history to the respondent and get his/her confirmation before writing it below.*<br>**Excuse me for a few minutes while I write out what you have told me. During this time, you are free to do something else for a few minutes. Afterwards, I have just a few more questions for you and we will close the interview. I appreciate your patience.**<br>*Please put in chronological order the key symptoms, any treatments received, and probing questions. Remember your goal is NOT to arrive at a diagnosis (that will be done by the central doctors) but the KEY symptoms and the KEY sickness history in chronological order.*<br>____			Minimum of 300 characters to be entered in the narrative text box<br><br>Recording ends after completion of this question.
**9001**	**Did (s)he receive any treatment for the illness that led to death?**	1. Yes<br>2. No<br>9. Doesn't know	 → 9011<br>→ 9011	
**9004**	**Did (s)he receive (or need) a blood transfusion?**	1. Yes<br>2. No<br>9. Doesn't know		
**9005**	**Did (s)he receive (or need) treatment/food through a tube passed through the nose?**	1. Yes<br>2. No<br>9. Doesn't know		
**9006**	**Did (s)he receive (or need) injectable antibiotics?**	1. Yes<br>2. No<br>9. Doesn't know		
**9008**	**Did (s)he receive (or need) an operation for the illness?**	1. Yes<br>2. No<br>9. Doesn't know		

**9011**	**Did (s)he receive any immunizations?**	1. Yes<br>2. No<br>9. Doesn't know	 → 9015<br>→ 9015	
**9012**	**Do you have the child's vaccination card?**	1. Yes<br>2. No	 → 9015	
**9013**	**Can I see the vaccination card?**	1. Yes<br>2. No	 → 9015	
**9014**	[Select EPI vaccines done.]	1. BCG<br>2. DPT 1,2,3<br>3. Hep B<br>4. Hib<br>5. Meningitis<br>6. Penta 1,2,3<br>7. Pneumo<br>8. Polio 1,2,3<br>10. Rota<br>11. No vaccines<br>99. Doesn't know		multiselect
**9015**	**Was care sought outside the home while (s)he had this illness?**	1. Yes<br>2. No<br>9. Doesn't know	 → 9018<br>→ 9018	
**9016**	**Where or from whom did you seek this care?**<br>*Enter more than one if applicable.*	1. Government Hospital<br>2. Government Health centre or clinic<br>3. Private Hospital<br>4. Ayush (Ayurveda, Unani, etc.)<br>5. Relative, friend (outside household)<br>6. Other<br>9. Doesn't know		multiselect
**9018**	**Did a health care worker tell you the cause of death?**	1. Yes<br>2. No<br>9. Doesn't know	 → 9034<br>→ 9034	
**9019**	**What did the health care worker say?**	Text		
**9034**	**Over the course of illness, did the total costs of care and treatment interfere with other routine household expenses?**	1. Yes<br>2. No<br>9. Doesn't know		
**9036**	**Was the death registered in the local government system (municipal or civil registration)?**	1. Yes<br>2. No<br>9. Doesn't know		

**9037**	**Did you receive a doctor's death certificate with the cause of death?**	1. Yes<br>2. No<br>9. Doesn't know	 → 12098<br>→ 12098	
**9038**	**Can I see the death certificate?**	1. Yes<br>2. No	 → 12098	
**9038a**	[Enter the cause of death.]			text
**9038b**	[Enter the date on the certificate.]	1. Day __ __<br>2. Month __ __<br>3. Year __ __ __ __		1- [1:31]<br>2- [1:12]<br>3- [2019:2022]
**Before I conclude, two short questions for you:**				
**12098**	**Would you say the interview was:**	1. Too long<br>2. Too short<br>3. About right length<br>9. No comment		Select- only one
**12099**	**Do you have any suggestions on how we can do better?**			text,maxlength[200]
NOTE: THE FOLLOWING QUESTIONS ARE FOR AUTO-POPULATION TO COMPLETE AND ARE NOT TO BE ASKED TO RESPONDENTS.				
**13004**	[Name of VA interviewer]	Text		
**13005**	[Time at start of interview]	[hh: __ __mm __ __]		
**13006**	[Date of interview]	[dd/mm/yyyy]		
**13021**	[Time at end of interview]	[hh: __ __mm __ __]		
